# Haploidentical hematopoietic cell transplantation with or without an unrelated cord blood unit for adult acute myeloid leukemia: a multicenter, randomized, open-label, phase 3 trial

**DOI:** 10.1038/s41392-024-01820-5

**Published:** 2024-05-06

**Authors:** Biqi Zhou, Jia Chen, Tianhui Liu, Yishan Ye, Yanming Zhang, Yiyang Ding, Hong Liu, MingQing Zhu, Xiao Ma, Xiaoli Li, Longfei Zhao, Zhihong Lin, He Huang, Yang Xu, Depei Wu

**Affiliations:** 1 National Clinical Research Center for Hematologic Diseases, Jiangsu Institute of Hematology, The First Affiliated Hospital of Soochow University, Suzhou, China; 2 Institute of Blood and Marrow Transplantation, Collaborative Innovation Center of Hematology, Soochow University, Suzhou, China; 3 Bone Marrow Transplantation Center, The First Affiliated Hospital, Zhejiang University School of Medicine, Hangzhou, Zhejiang China; 4 Department of Hematology, The Affiliated Huai’an Hospital of Xuzhou Medical University and The Second People’s Hospital of Huai’an, Huai’an, China; 5 Soochow Hopes Hematology Hospital, Suzhou, China; 6 Department of Hematology, Hygeia Suzhou Yongding Hospital, Suzhou, China

**Keywords:** Haematological cancer, Clinical trials

## Abstract

Coinfusion of unrelated cord blood (UCB) units in haploidentical hematopoietic cell transplantation (haplo-HCT) (haplo-cord HCT) for hematopoietic malignancies showed promising results in previous reports, but the efficiency of haplo-cord HCT in acute myeloid leukemia (AML) still lacks sufficient evidence. This multicenter, randomized, phase 3 trial (ClinicalTrials.gov NCT03719534) aimed to assess the efficacy and safety of haplo-cord HCT in AML patients. A total of 268 eligible patients aged 18–60 years, diagnosed with measurable residual disease in AML (excluding acute promyelocytic leukemia), with available haploidentical donors and suitable for allotransplantation, were randomly allocated (1:1) to receive haplo-cord HCT (*n* = 134) or haplo-HCT (*n* = 134). The 3-year overall survival (OS) was the primary endpoint in this study. Overall median follow-up was 36.50 months (IQR 24.75–46.50). The 3-year OS of Haplo-cord HCT group was better than haplo-HCT group (80.5%, 95% confidence interval [CI]: 73.7–87.9 vs. 67.8% 95% CI 60.0–76.5, *p* = 0.013). Favorable progression-free survival (70.3%, 95% CI 62.6–78.8 vs. 57.6%, 95% CI 49.6–67.0, *p* = 0.012) and cumulative incidence of relapse (12.1%, 95% CI 12.0–12.2 vs. 30.3%, 95% CI 30.1–30.4, *p* = 0.024) were observed in haplo-cord HCT group. Grade 3–4 adverse events (AEs) within two years posttransplantation in the two groups were similar. Haplo-cord HCT patients exhibited a faster cumulative incidence of neutrophil recovery (*p* = 0.026) and increased T-cell reconstitution in the early period posttransplantation. Haplo-cord HCT can improve OS in AML patients without excessive AEs, which may exert additional benefits for recipients of haplo-HCT.

## Introduction

Acute myeloid leukemia (AML) is the most prevalent leukemia among adults and responsible for the highest proportion (62%) of deaths related to leukemia.^1^ Despite advancements in intensive induction chemotherapy, hypomethylating agents and targeted medications improving the outcomes of AML patients, allogeneic hematopoietic cell transplantation (allo-HCT) remains a critical treatment option due to its high curative potential.^2,3^ Allo-HCT had anti-leukemic effects through a pretransplant conditioning regimen and posttransplant graft-versus-leukemia (GVL) response primarily mediated through alloreactive lymphocytes.^4^

Since available human leukocyte antigen (HLA)-matched donors are scarce, haploidentical hematopoietic cell transplantation (haplo-HCT) has been developed and widely used as alternative hematopoietic cell transplantation (HCT). Haplo-HCT is considered to have potent GVL effects accompanied by increased risk of graft-versus-host disease (GVHD) and treatment-related mortality.^5,6^ It is reported that younger, male, non-inherited maternal antigen (NIMA)-mismatched donors are potential factors associated with reduced risk of GVHD.^7,8^ Beyond refining donor selection criteria, advancements in understanding hematopoietic cell sources (such as bone marrow grafts) and GVHD prophylaxis (such as the use of anti-thymocyte globulin [ATG] and posttransplant cyclophosphamide) have effectively mitigated the risk of GVHD.^7,9^ However, the cumulative incidence of relapse (CIR) after haplo-HCT remains unsatisfactory. According to an international registry study, 32% of AML patients experienced relapse posttransplantation.^10^ Notably, posttransplant death is mainly caused by relapse either within +100 days or beyond +100 days posttransplantation, which accounts for 60% of deaths after haplo-HCT.^11^ Research findings indicated that killer immunoglobulin-like receptor (KIR) ligand match could promote NK-cell recovery and thus reduce relapse.^12^ Adding decitabine and recombinant human granulocyte colony-stimulating factor (rhG-CSF) in the conditioning regimen of haplo-HCT also resulted in rapid recovery of T-cell and NK-cell and a 23% decrease of 2-year CIR.^13^ Besides, the combination of chimeric antigen receptor (CAR)-T cell therapy with transplantation, along with posttransplantation maintenance strategies (such as venetoclax and sorafenib), shows promise in preventing disease relapse in AML patients.^14^

Unrelated cord blood (UCB) is another common alternative hematopoietic cell source. The restricted limited cell numbers of UCB have constrained its utilization only to children and adults of smaller stature. Also, cord blood transplant (CBT) usually leads to result in delayed engraftment and compromised immune reconstitution, which results in a high rate of nonrelapse mortality (NRM).^15,16^ However, patients with CBT are reported to have a promising GVL effect and mild GVHD. In efforts to enhance transplantation strategies, a number of research groups have explored coinfusion of a haploidentical graft and a UCB unit in patients with malignant hematological diseases over the past decades, hoping to preserve the benefits of both grafts while circumventing their drawbacks.^17–22^ In 2014, we reported a single-arm prospective study of malignant hematological disease patients who had unmanipulated haploidentical grafts and UCB units coinfused, showing improved outcomes when compared with the historical data of our center.^17,23^ Accelerated platelet recovery, improved overall survival (OS), enhanced disease-free survival and better GVHD-free, relapse-free survival were observed in relapsed/refractory hematologic malignancies patients who received haplo-cord HCT compared to those who received CBT.^24^ When compared to haplo-HCT, several retrospective studies have reported significantly improved OS, reduced relapse and similar cumulative incidence of GVHD in acute leukemia or myelodysplastic syndromes patients who received haplo-cord HCT.^25,26^ Similarly, our previous research in B-cell acute lymphoblastic leukemia (B-ALL) showed promoted T-cell reconstitution and improved prognoses after coinfusion of UCB unit in haplo-HCT.^27^ However, although coinfusion of unmanipulated haploidentical grafts and UCB units showed promising outcomes in hematological diseases, the efficacy and safety of haplo-cord HCT in the predominant adult leukemia, AML, have not been clarified. To date, no prospective randomized results comparing haplo-cord HCT and haplo-HCT for AML patients have been reported. Also, the reports related to immune reconstitution patterns after haplo-cord HCT are rare.

In the current report, we reported the first analysis results from a multicenter, randomized phase 3 trial to assess the efficacy and safety of coinfusion of UCB units for AML patients undergoing haplo-HCT.

## Results

### Patients

**Fig. 1:**
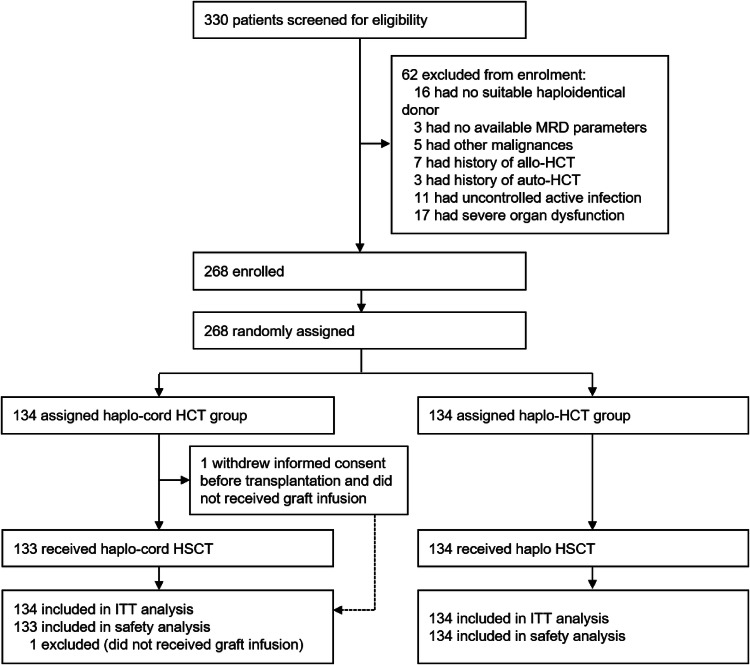
Trial flowchart

Between June 1, 2017, and June 30, 2021, 330 candidates aged 18–60 with AML preparing to undergo haplo-HCT were consecutively screened for eligibility. Ultimately, 268 patients were enrolled and randomly allocated to receive a coinfusion unrelated UCB unit in addition to haploidentical allograft versus haploidentical allograft alone (Fig. 1). After randomization, one haplo-cord HCT patient withdrew the informed consent before conditioning and refused transplantation therapy. This patient was excluded from the safety analysis. All donors and recipients were East Asian. Among the 268 recipients, the median age was 39 years (IQR 29–47), and 120 (44.8%) of them were female. The haplo-cord HCT and haplo-HCT groups had similar baseline characteristics (Table 1). The median count of infused haploidentical CD34^+^ cells was 4.03 × 10^6^/kg (IQR 3.32–5.10) and 4.17 × 10^6^/kg (IQR 3.21–5.83) in the haplo-cord HCT and haplo-HCT cohorts, respectively. The median dose of infused UCB CD34^+^ cells was 4.60 × 10^4^/kg (IQR 1.02–17.41) in the haplo-cord HCT recipients.

**Table 1:** Baseline characteristics

	Haplo-cord HCT	Haplo-HCT
	*N* = 134	*N* = 134
**Sex, male/female**	75/59	73/61
**Age, years**	39.00 (30.00–45.00)	38.50 (29.00–48.75)
**2022 ELN Risk Category**		
Adverse	46 (34.3%)	44 (32.8%)
Intermediate	73 (54.5%)	68 (50.7%)
Favorable	6 (4.5%)	7 (5.2%)
Unknown	9 (6.7%)	15 (11.2%)
**Detected at diagnosed**		
White blood cell, ×10^9^/L	14.76 (3.70–40.04)	15.14 (3.06–56.77)
Bone marrow blast, %	60.00 (44.00–78.13)	63.42 (40.50–81.00)
No. of induction courses		
≤2	114 (85.1%)	111 (82.8%)
> 2	20 (14.9%)	23 (17.2%)
Extramedullary leukemia	6 (4.5%)	4 (3.0%)
**Detected at randomization**		
Interval from diagnosis to randomization, months	5.00 (4.00–7.50)	5.50 (4.50–7.88)
Disease status		
CR1	90 (67.2%)	94 (70.1%)
CR2/CR3	26 (19.4%)	23 (17.2%)
PR/NR	18 (13.4%)	17 (12.7%)
Haploidentical hematopoietic cell source^a^		
BM	6 (4.5%)	2 (1.5%)
PB	68 (51.1%)	82 (61.2%)
Combination of BM and PB	59 (44.4%)	50 (37.3%)
Haploidentical grafts^a^		
Mononuclear cells, ×10^8^/kg	9.48 (7.55–12.35)	10.20 (7.24–14.62)
CD34^+^ cells, ×10^6^/kg	4.03 (3.32–5.10)	4.17 (3.21–5.83)
Donnor type		
Parent	33 (24.8%)	34 (25.4%)
Offspring	69 (51.9%)	63 (47.0%)
Sibling	31 (23.3%)	37 (27.6%)
Donnor sex, male/female	89/44	95/39
Donnor age, years	29.00 (22.00–43.75)	27.00 (15.00–45.25)
Unrelated cord blood grafts^a^		
Viability post-thawing, %	90.00 (87.50–94.75)	NA
Mononuclear cells^b^, ×10^6^/kg	16.59 (1.73–31.71)	NA
CD34^+^ cells^b^, ×10^4^/kg	4.60 (1.02–17.41)	NA

*HCT* hematopoietic cell transplantation, *PR* partial response, *NR* nonresponse

Data are the median (IQR) or n (%).

^a^One haplo-cord HCT patient who withdrew informed consent and did not receive transplantation therapy was not included

^b^Numbers of UCB cells infused after resuscitation

### Overall survival

**Fig. 2:**
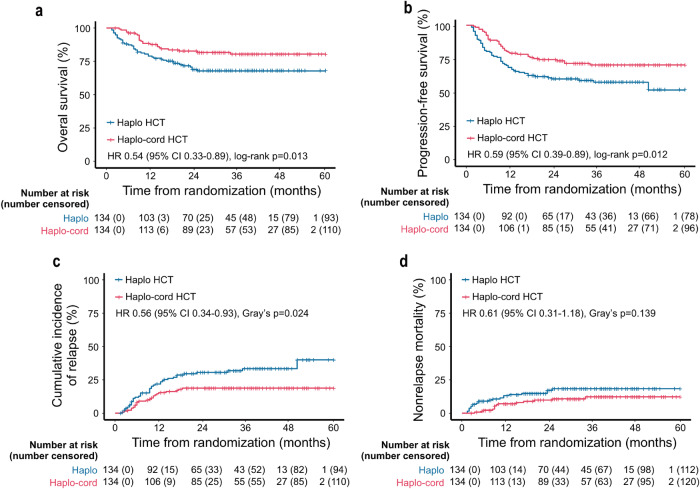
Prognoses of primary and secondary outcomes of the haplo-cord HCT and haplo-HCT groups in the ITT population. **a** Kaplan‒Meier plots of OS. **b** Kaplan‒Meier plots of PFS. **c** Cumulative incidence curves of CIR. **d** Cumulative incidence curves of the NRM

This trial followed participants for a median duration of 36.5 months (IQR 24.75–46.50) from randomization. The 3-year OS in haplo-cord HCT and haplo-HCT recipients were 80.5% (95% confidence interval [CI] 73.7–87.9) and 67.8% (95% CI 60.0–76.5), respectively (hazard ratio [HR] 0.54, 95% CI 0.33–0.89, log-rank *p* = 0.013) (Fig. 2a). The median OS of either group was not reached. Similar improvement was found with regard to the 3-year OS of the patients who underwent haplo-cord HCT in the population who received family donors with/without UCB grafts infusion (81.1%, 95% CI 74.4–88.5 vs. 67.8%, 95% CI 60.0–76.5, HR 0.52, 95% CI 0.31–0.86, log-rank *p* = 0.009) (Supplementary Fig. 1a). During follow-up, 24 (17.9%) haplo-cord HCT and 41 (30.6%) haplo-HCT patients died. The causes of death are listed in Supplementary Table 2. Among the patients who died, no difference in causes of death was observed between the two groups.

A consistent pattern of improved 3-year OS with haplo-cord HCT was noted across multiple subgroups (Supplementary Fig. 1b). Additionally, haplo-cord HCT and haplo-HCT showed similar outcomes in patients with partial response or nonresponse at randomization (Supplementary Fig. 2a). In patients who achieved complete remission (CR) as well as measurable residual disease (MRD)-response at randomization, limited benefit without a significant difference from haplo-cord HCT was observed (Supplementary Fig. 2b). In patients who achieved CR without MRD-response at randomization, coinfusion of UCB significantly improved 3-year outcomes (OS: 82.2%, 95% CI 71.2–92.3 vs. 61.1%, 95% CI 47.9–77.9, HR 0.44, 95% CI 0.21-0.94, log-rank *p* = 0.029; progression-free survival [PFS]: 74.2%, 95% CI 64.0–86.0 vs. 47.2%, 95% CI 34.9–64.0, HR 0.42, 95% CI 0.22–0.79, log-rank *p* = 0.004; CIR: 17.1%, 95% CI 16.8–17.4 vs. 35.7%, 95% CI 35.2–36.2, HR 0.36, 95% CI 0.16–0.81, Gray’s *p* = 0.011) (Supplementary Fig. 2c). Multivariable analysis in the intention-to-treat (ITT) population showed that coinfusion of UCB was the only independent protective factor for OS; induction chemotherapy >2 courses and infection posttransplantation were independent predictive factors for decreased 3-year OS (Table 2).

**Table 2:** Univariate and multivariate analyses of outcomes

	Univariate analysis			Multivariate analysis		
	HR	95% CI	*P*	HR	95% CI	*P*
**OS**						
Sex, male vs. female	0.83	0.50–1.36	0.457	–	–	–
Age, years	1.01	0.99–1.03	0.395	–	–	–
2022 ELN adverse risk, yes vs. no	1.05	0.62–1.78	0.859	–	–	–
White blood cell at diagnosis, ≥30 × 10^9^/L vs. <30 × 10^9^/L	1.68	1.01–2.78	0.046	1.53	0.91–2.58	0.109
Induction chemo, >2 vs. ≤2	2.26	1.28–3.98	0.005	2.06	1.10–3.85	0.023
Extramedullary leukemia, yes vs. no	2.82	1.13–7.04	0.026	2.00	0.71–5.62	0.191
CR1 at randomization, yes vs. no	0.60	0.36–0.98	0.042	1.013	0.59–1.74	0.964
Interval from diagnosis to randomization, months	1.02	0.99–1.05	0.306	-	-	-
Coinfusion of UCB, yes vs. no	0.54	0.33–0.89	0.016	0.43	0.25–0.76	0.004
Infections^a^, yes vs. no	3.69	2.21–6.17	<0.001	3.47	1.98–6.08	<0.001
Grade II-IV aGVHD, yes vs. no	2.17	1.27–3.70	0.005	1.64	0.90–2.99	0.110
Moderate/severe cGVHD, yes vs. no	2.45	1.30–4.63	0.006	1.37	0.68–2.78	0.384
Center			0.238			
**DFS**						
Sex, male vs. female	0.87	0.58–1.31	0.512	–	–	–
Age, years	1.01	0.99–1.02	0.582	–	–	–
2022 ELN adverse risk, yes vs. no	1.34	0.88–2.06	0.174	–	–	–
White blood cell at diagnosis, ≥30 × 10^9^/L vs. <30 × 10^9^/L	1.20	0.78–1.85	0.413	–	–	–
Induction chemo, >2 vs. ≤2	3.08	1.96–4.83	<0.001	2.95	1.86–4.68	<0.001
Extramedullary leukemia, yes vs. no	1.89	0.77–4.65	0.168	–	–	–
CR1 at randomization, yes vs. no	0.52	0.35–0.78	0.002	0.64	0.41–0.98	0.039
Interval from diagnosis to randomization, months	1.01	0.99–1.04	0.374	–	–	–
Coinfusion of UCB, yes vs. no	0.60	0.39–0.91	0.016	0.49	0.32–0.75	0.001
Infections^a^, yes vs. no	1.69	1.13–2.53	0.011	1.41	0.92–2.16	0.113
Grade II-IV aGVHD, yes vs. no	1.58	0.98–2.54	0.062	1.43	0.88–2.33	0.149
Moderate/severe cGVHD, yes vs. no	2.01	1.13–3.58	0.018	1.45	0.81–2.60	0.217
Center			0.419			
**CIR**						
Age, years	0.67	0.40–1.12	0.120	–	–	–
Age, years	0.99	0.97–1.02	0.620	–	–	–
2022 ELN adverse risk, yes vs. no	1.26	0.76–2.10	0.370	–	–	–
White blood cell at diagnosis, ≥30 × 10^9^/L vs. <30 × 10^9^/L	1.50	0.90–2.49	0.120	–	–	–
Induction chemo, >2 vs. ≤2	2.87	1.69–4.86	<0.001	2.72	1.55–4.76	<0.001
Extramedullary leukemia, yes vs. no	1.46	0.41–5.24	0.560	–	–	–
CR1 at randomization, yes vs. no	0.51	0.31–0.84	0.0083	0.56	0.34–0.93	0.026
Interval from diagnosis to randomization, months	1.01	0.98–1.05	0.490	–	–	–
Coinfusion of UCB, yes vs. no	0.57	0.34–0.94	0.029	0.51	0.31–0.86	0.010
Infections^a^, yes vs. no	1.47	0.90–2.39	0.120			
Grade II-IV aGVHD, yes vs. no	0.66	0.33–1.35	0.260	–	–	–
Moderate/severe cGVHD, yes vs. no	0.57	0.27–1.20	0.140	–	–	–
Center			0.910			
**NRM**						
Sex, male vs. female	1.28	0.67–2.45	0.460	–	–	–
Age, years	1.02	0.99–1.06	0.160	–	–	–
2022 ELN adverse risk, yes vs. no	1.29	0.65–2.57	0.460	–	–	–
White blood cell at diagnosis, ≥30 × 10^9^/L vs. <30 × 10^9^/L	1.09	0.53–2.22	0.820	–	–	–
Induction chemo, >2 vs. ≤2	1.86	0.85–4.08	0.120	–	–	–
Extramedullary leukemia, yes vs. no	2.83	0.84–9.49	0.092	1.96	0.41–9.47	0.400
CR1 at randomization, yes vs. no	0.66	0.34–1.29	0.230	–	–	–
Interval from diagnosis to randomization, months	1.03	0.99–1.07	0.130	–	–	–
Coinfusion of UCB, yes vs. no	0.61	0.31–1.18	0.140	–	–	–
Infections^a^, yes vs. no	5.72	2.69–12.20	<0.001	3.60	1.62–8.03	0.002
Grade II-IV aGVHD, yes vs. no	4.14	2.14–8.00	<0.001	3.18	1.57–6.46	0.001
Moderate/severe cGVHD, yes vs. no	1.89	0.94–3.84	0.076	1.25	0.55–2.85	0.600
Center			0.170			

^a^More than grade 3 CTCAE

### Secondary efficacy outcomes

A total of 64 patients experienced relapse by the time of the statistical analysis (on Dec 2, 2022), including 24 haplo-cord HCT patients and 40 haplo-HCT patients. The 3-year PFS was 70.3% (95% CI 62.6–78.8) in haplo-cord HCT patients and 57.6% (95% CI 49.6–67.0) in haplo-HCT patients (HR 0.59, 95% CI 0.39–0.89, log-rank *p* = 0.012) (Fig. 2b). Haplo-cord HCT recipients showed markedly lower 3-year CIR compared to haplo-HCT recipients (12.1%, 95% CI 12.0–12.2 vs. 30.3%, 95% CI 30.1–30.4, HR 0.56, 95% CI 0.34–0.93, Gray’s *p* = 0.024) (Fig. 2c). Comparable 3-year NRM was observed in the two groups (14.0%, 95% CI 13.9–14.1 vs. 17.3%, 95% CI 17.2–17.4, HR 0.61, 95% CI 0.31–1.18, Gray’s *p* = 0.139) (Fig. 2d). In multivariate analysis, coinfusion of UCB, induction chemotherapy >2 courses and CR1 at randomization were independent parameters associated with PFS. Additionally, induction chemotherapy >2 courses could be independently indicative of unfavorable prognosis, whereas coinfusion of UCB and CR1 at randomization were independent protective factors for CIR. Infections and grade II-IV Acute GVHD (aGVHD) were found to be independent risk factors for unfavorable NRM (Table 2).

### Safety

Table 3 summarizes adverse events (AEs) that occurred from enrollment until two years posttransplantation. Patients who underwent haplo-cord HCT showed more grade 1 or 2 general disorders and administration site condition AEs (64 [48.1%] and 24 [17.9%]), especially for infusion-related reactions such as headache and hypertension at the time of UCB infusion. Sixty-seven (50.4%) of 133 haplo-cord HCT patients and 63 (47.0%) of 134 haplo-cord HCT patients experienced at least one of grade 3 or 4 AE. Infections (45 [33.8%] and 39 [29.1%]), aGVHD (27 [20.3%] and 17 [12.7%]) and chronic GVHD (cGVHD) (16 [12.0%] and 22 [16.4%]) were the most commonly reported grade 3 or 4 AEs among patients in both two groups. Eleven (8.3%) patients who underwent haplo-cord HCT and 20 (14.9%) patients who underwent haplo-HCT died from AEs. The most frequent serious AEs in both two groups were infections (20 [15.0%] and 22 [16.4%]), aGVHD (20 [15.0%] and 19 [14.2%]) and cGVHD (11 [8.3%] and 12 [8.9%]).

**Table 3:** Nonhematological adverse events irrespective of causality

	Haplo-cord HCT (*n* = 133)				Haplo-HCT (*n* = 134)			
	Grade 1-2	Grade 3	Grade 4	Grade 5	Grade 1-2	Grade 3	Grade 4	Grade 5
Cardiac	20 (15.0)	1 (0.8)	1 (0.8)	1 (0.8)	18 (13.4)	1 (0.7)	2 (1.5)	0
Gastrointestinal^a^	66 (49.6)	16 (12.0)	0	0	71 (53.0)	14 (10.4)	0	0
General disorders and administration site conditions	64 (48.1)	0	0	0	24 (17.9)	0	0	0
Hepatobiliary or pancreatic^a^	4 (3.1)	0	1 (0.8)	0	5 (3.7)	0	1 (0.7)	0
Immune system	10 (7.5)	0	0	0	7 (5.2)	1 (0.7)	0	0
Infections^b^	18 (13.5)	32 (24.1)	13 (9.8)	5 (3.8)	20 (14.9)	27 (20.1)	12 (9.0)	9 (6.7)
Nervous system	19 (14.3)	2 (1.5)	1 (0.8)	0	17 (12.7)	1 (0.7)	1 (0.7)	1 (0.7)
Renal or genitourinary	30 (22.6)	7 (5.3)	1 (0.8)	0	18 (13.4)	7 (5.2)	0	0
Skin and subcutaneous tissue^a^	15 (11.3)	2 (1.5)	0	0	13 (9.7)	3 (2.2)	0	0
Vascular	10 (7.5)	2 (1.5)	0	0	9 (6.7)	2 (1.5)	0	0
aGVHD	18 (13.5)	11 (8.3)	16 (12.0)	1 (0.8)	13 (9.7)	9 (6.7)	8 (6.0)	6 (4.5)
cGVHD	19 (14.3)	9 (6.8)	7 (5.3)	3 (2.3)	20 (14.9)	14 (10.4)	8 (6.0)	2 (1.5)

The table shows grade 1-2 adverse events that occurred in more than 10% of patients and all grade 3, 4, and 5 adverse events, which were recorded from enrollment to 2 years post-transplantation. Data are the n (%)

^a^Excluded patients with GVHD

^b^Excluded patients with cytomegalovirus viraemia and Epstein‒Barr virus viraemia

**Fig. 3:**
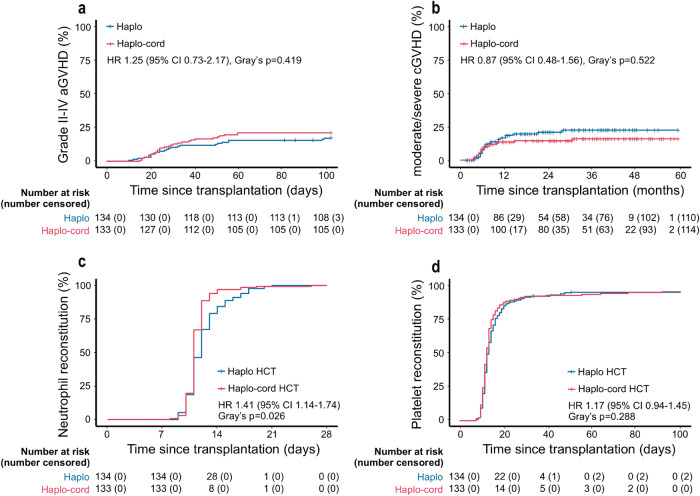
GVHD and hematopoietic recovery of the haplo-cord HCT and haplo-HCT groups. **a** Cumulative incidence curves of grade II-IV aGVHD. **b** Cumulative incidence curves of moderate/severe cGVHD. **c** Cumulative incidence curves of neutrophil recovery. **d** Cumulative incidence curves of platelet recovery

Interestingly, although post- hoc analysis revealed cumulative incidences of grade II-IV aGVHD in the two groups (Fig. 3a), lower stage 4 intestinal aGVHD rate was observed in haplo-cord HCT patients (3 [14.3%] and 8 [61.5%]) (Supplementary Table 3). No significant variance was detected in cumulative rates of moderate/severe cGVHD, infections, cytomegalovirus (CMV) viremia or Epstein‒Barr virus (EBV) viremia between the two groups. Nevertheless, the 2-year incidence of hemorrhagic cystitis (HC) was elevated in the haplo-cord HCT cohort (25.8%, 95% CI 25.7–26.0 vs. 9.7%, 95% CI 9.7–9.8, HR 2.27, 95% CI 1.19–4.36, Gray’s *p* = 0.011) (Fig. 3b, Supplementary Fig. 3).

### MRD response

In patients with advanced diseases or without MRD-response at randomization, 72.8% (59/81) of haplo-cord HCT and 71.4% (45/63) of haplo-HCT patients attained MRD-response at the first month posttransplantation (*p* = 0.551). In patients with MRD-response at the first-month posttransplantation, the haplo-cord HCT group showed significantly less MRD relapse (loss of MRD-response) than the haplo-HCT group at three months and six months posttransplantation (+3 months: 6.6% [7/106] and 17.9% [17/95], *p* = 0.014; +6 months: 6.3% [6/96] and 16.1% [14/87], *p* = 0.033).

### Chimerism and hematopoietic engraftment

Within the first 100 days posttransplantation, 121 (93.8%) of 129 haplo-cord HCT recipients (except 2 patients relapsed and 1 patient died) and 119 haplo-HCT recipients (except 6 patients relapsed and 9 patients died) achieved complete haploidentical engraftment. The chimerism level of haploidentical graft in bone marrow (BM) were comparable between both groups (+1 month: 98.6%, IQR 98.1–98.9 vs. 98.6%, IQR 98.3–99.0, *p* = 0.396; +3 months: 98.6%, IQR 98.3–99.0 vs. 98.5%, IQR 98.2–98.9, *p* = 0.114). Interestingly, microchimerism of UCB at month 1 was detected in the haplo-cord HCT group using next-generation sequencing (NGS) in BM (0.30%, IQR 0.04–1.60), and the chimerism percentage of UCB decreased gradually as time passed (+3 months: 0.05%, IQR 0.03–0.09; +6 months: 0.00%, IQR 0.00–0.02). A similar trend was also observed in peripheral blood (PB) (+14 days: 1.91%, IQR 0.58–2.84; +1 month: 0.06%, IQR 0.03–0.40; +3 months: 0.00%, IQR 0.00–0.04) (Supplementary Fig. 4). One haplo-cord HCT recipient exhibited complete UCB engraftment and seven recipients showed mixed chimerism (Supplementary Table 4).

All patients exhibited neutrophil engraftment within 28 days posttransplantation, with a significantly shorter median recovery time after haplo-cord HCT (11 days, IQR 11–12 days) compared to haplo-HCT (12 days, IQR 11–13 days, *p* = 0.001). Also, the neutrophil recovery incidence differed significantly between the two groups (HR 1.41, 95% CI 1.14–1.74, Gray’s *p* = 0.026) (Fig. 3c). There were no statistical differences found in the median recovery time of platelet (13 days, IQR 12–15 days vs. 13 days, IQR 12–16 days, *p* = 0.651) or the platelet recovery incidence between patients undergoing haplo-cord HCT and haplo-HCT (Fig. 3d).

### Patterns of immune reconstitution

Immune reconstitution results were detected in 45 haplo-cord HCT and 21 haplo-HCT recipients with complete haploidentical chimerism after transplantation who agreed to additional collection of PB samples. Rapid NK-cell reconstitution and delayed recovery of B cells were observed in both groups (Supplementary Table 5; Supplementary Fig. 5a, b), and the rules of reconstitution were similar in the two groups in these cells. T-cell numbers gradually increased during the first three months posttransplant (Supplementary Fig. 5c). Notably, a faster T-cell increase occurred in the early period posttransplantation and had a higher T-cell count at month 1 posttransplantation than the haplo-HCT group (0.13 × 10^9^/L, IQR 0.08–0.33 vs. 0.06 × 10^9^/L, IQR 0.03–0.09, *p* = 0.034).

Recovery of T-cell subsets at month 1 posttransplantation was further assessed (Supplementary Fig. 6). The haplo-cord HCT group had higher CD4^+^ T-cell counts (*p* = 0.024) and CD8^+^ T-cell counts (*p* = 0.044) compared to the haplo-HCT group. Elevated HLA-DR^+^CD4^+^ T-cell (*p* = 0.048), Ki67^+^CD4^+^ T-cell (*p* = 0.036) and Ki67^+^CD8^+^ T-cell (*p* = 0.046) counts were also observed in the haplo-cord HCT group. Additionally, the haplo-cord HCT group had higher levels of CD4^+^ T_em_ cells (*p* = 0.042), T_reg_ cells (*p* = 0.045), Th2 cells (*p* = 0.026), Th17 cells (*p* = 0.047), CD8^+^ T_em_ cells (*p* = 0.047), and Tc2 (*p* = 0.045) cells.

## Discussion

This randomized trial (NCT03719534) showed that coinfusion of UCB units could enhance OS in adult AML patients undergoing haplo-HCT. Additionally, haplo-cord HCT patients also had improved PFS and CIR, and their NRM was similar to that of haplo-HCT patients. Safety benchmarks, including grade 3 or worse AEs within two years posttransplantation, showed no significant differences between the two study groups.

In 2001, *MN Fernández* et al. first reported early granulocyte engraftment after CBT combined with purified haploidentical CD34^+^ cells coinfusion in three patients.^22^ Thereafter, several research groups all over the world also attempted transplantation strategy combined with haploidentical and UCB grafts.^17–22^ *Dr. M Kwon* et al. and *Dr. Koen van Besien* et al. reported that haplo-cord HCT had faster neutrophil engraftment, lower GVHD rates and similar prognoses to haplo-HCT after myeloablative or reduced-intensity conditionings.^18,19^ In these studies, patients were infused with UCB grafts and magnetic bead-enriched haploidentical CD34^+^ cells. Most patients had mixed chimerism within the first 100 days posttransplantation, which was superseded by stable cord blood engraftment. The haploidentical cells acted as transient bridging grafts throughout neutropenia after transplantation^18–22,28–30^ While in patients infused with unmanipulated haploidentical grafts and UCB units, >90% of patients showed completed haploidentical engraftment.^17,25,27,31–33^ Interestingly, microchimerism of the UCB graft was detected in the earlier period posttransfusion despite a decreasing trend over time in this trial. The results of chimerism were opposed to the research coinfused UCB units and haploidentical CD34^+^ cells. It should be noticed that in studies exhibiting complete haploidentical engraftment, the haploidentical grafts were T-cell-replete, which might result in a stronger engraftment ability.^17,34^ The differing cell counts in the UCB units and haploidentical grafts might also contribute to different outcomes.^35^

In comparison to haplo-HCT, T-cell-replete haplo-cord HCT exhibited comparable GVHD rates and significantly improved survival.^25,31^ Our previous retrospective research found dramatically improved OS and reduced relapse rates after haplo-cord HCT in B-ALL patients.^27^ In this multicenter randomized controlled trial, haplo-cord HCT accelerated neutrophil recovery and improved survival in AML patients. Additionally, haplo-cord HCT patients experienced less molecular relapse within the period of detectable UCB graft microchimerism. The presence of MRD posttransplantation is related to a higher relapse rate and unfavorable survival.^36,37^ Significant improvement in 3-year CIR was also observed in this trial, especially in patients who achieved CR without MRD-response pretransplant. Multivariate analysis confirmed that in AML patients after haplo-HCT, coinfusion of UCB was independently associated with favorable 3-year OS, PFS and CIR. These results suggest that coinfusion of UCB units can enhance the GVL effect in haplo-HCT. This T-cell-replete haplo-cord HCT had better OS, lower relapse and II-IV grade aGVHD, and similar cGVHD compared to previous CBT reports,^38–40^ However, it still lacks evidence from multicenter, prospective studies.

Early immune recovery posttransplantation is reported to be associated with a significant GVL effect.^41^ In this study, haplo-cord HCT patients showed faster T-cell reconstitution in the early posttransplantation period in comparison to haplo-HCT patients. Cord blood grafts are considered to provide superior antitumor effects and reduce relapse.^15,42^ Furthermore, mismatches of HLA comparisons between UCB units and haploidentical grafts were related to recipient outcomes, suggesting that in addition to antitumor effects directly mediated by UCB, there might be other mechanisms for enhancing the GVL effect.^25,31^ In this trial, microchimerism of the UCB graft and differences in T-cell recovery earlier in the three months posttransfusion indicated that the graft-versus-graft (GVG) effect might exist in haplo-cord HCT. Regrettably, samples of different cell populations were not collected for chimerism detection. However, the median percent of UCB chimerism in PB in the first-month posttransplantation was much lower than the median percent of T cells among PB (0.06% vs. 3.47%), indicating that most T cells were haploidentical unit-derived. Additional research is needed to investigate the impact of coinfused UCB units on immune reconstitution and the GVL effect.

The GVL effect is usually accompanied by a potential risk of GVHD.^43^ Remarkably, coinfusion of UCB units in haplo-HCT reduced AML patient relapse, but it did not statistically impact cumulative incidences of grade II-IV aGVHD and cGVHD. Although haplo-cord HCT patients showed a slightly higher cumulative incidence of grade II-IV aGVHD than haplo-HCT patients, the rate of stage 4 intestinal aGVHD decreased, which resulted in fewer grade 5 aGVHD AEs. Immune reconstitution detection revealed increased CD4^+^ and CD8^+^ T-cell proliferation and differentiation, such as higher numbers of Th2, Th17, Treg and Tc2 cells. Studies have indicated that Tc2 and Th17 cells promote the GVL effect and that T_reg_ cells and Th2 cells can regulate GVHD without impairing alloengraftment.^44–46^ These findings suggested that coinfusion of UCB units might affect T-cell reconstitution and immune signaling, causing an increasing GVL effect without excessive GVHD risks.

In this study, grade 3 or worse AEs were comparable between the haplo-cord HCT and haplo-HCT groups, suggesting the safety of coinfusion of UCB in this population. It has been reported that CBT is associated with a high proportion of infection-related deaths within 100 days posttransplant.^47^ Moreover, HC is considered a significant complication in CBT patients.^48^ In this study, coinfusion of the UCB unit did not increase the cumulative incidence of infection. Additionally, haplo-cord HCT showed a significantly increased incidence of HC, which should be considered in the clinical process. These results are consistent with previous reports.^25,27,31^

This trial had some limitations. First, all the participants received a homogeneous protocol employing myeloablative conditioning with Bu/Cy and ATG, infused the UCB unit before the haploidentical graft, and the efficacy and safety of UCB coinfusion in other transplantation modalities remain to be examined. Second, this trial only enrolled 18–60 years old AML participants, and the efficacy and safety in other patients with haplo-cord HCT, such as elder AML patients, still need further studies. Third, the sample sizes in this trial might have low test power for subgroup analysis. Finally, it is difficult to conduct a blinded trial which results in inevitable biases favoring the experimental group.

In conclusion, this trial was the first to demonstrate that confusion of UCB units in haplo-HCT could improve OS for AML patients, which was mainly attributed to reduced risk of relapse. Additionally, it was observed that coinfusion of UCB enhanced the early recovery of T cells after haplo-HCT. The novel prognostic insights derived from our study support the safety and efficacy of haplo-cord HCT for AML patients, and the impact of coinfused UCB on immune reconstitution post-HCT deserves further investigation.

## Materials and methods

### Study design and participants

This was a multicenter, randomized, open-label, prospective trial carried out in five centers in China (Supplementary Table 1). The study has been registered on ClinicalTrials.gov under the identifier NCT03719534. The study received ethics committee approval at each participating center and followed the Declaration of Helsinki and the principles of Good Clinical Practice. Written informed consent was obtained from each participant before initiation of the study intervention. Agreement to additional collection of PB samples for immune reconstitution detection was also requested. The authors of this report had access to all study data.

Eligible candidates Patients should meet the following criteria: (1) aged 18–to 60; (2) diagnosed with AML based on the National Comprehensive Cancer Network criteria; (3) had available MRD parameters evaluated through flow cytometry (FCM) or quantitative polymerase chain reaction (qPCR); (4) had a compatible haploidentical donor available and willingness to receive haplo-HCT; (5) had Eastern Cooperative Oncology Group performance status between 0-to 3. Exclusion criteria were as follows: (1) had acute promyelocytic leukemia or other malignancies; (2) did not acquire a suitable UCB unit; (3) had a previous history of autologous or allogeneic HCT or chimeric antigen receptor T-cell therapy; (4) had uncontrolled infection or severe organ dysfunction; (5) were pregnant or lactating. Patients could exit the study at any time by withdrawing informed consent or at the investigator’s discretion.

### Randomization and masking

Study site staff evaluated patients for eligibility within seven days preceding randomization. Eligible patients were randomly assigned in a 1:1 ratio into two groups to receive either haplo-cord HCT or haplo-HCT. Randomization was done using permuted blocks of size four through an independent web-based system. The participants and investigators were aware of the treatment allocation. The data analysis was evaluated in a masked manner.

### Treatment interventions

All centers used the same criteria for selecting family donors and UCB units. Family donors were chosen based on their HLA typing results, age (younger preferred), sex (male preferred), and health status (better preferred). High-resolution HLA typing at HLA-A, HLA-B, HLA-C, HLA-DRB1 and HLA-DQ B1 loci was performed in all donor-recipient pairs. All recipients received grafts from family members who shared one HLA haplotype with the recipient but varied in the HLA antigens of the unshared HLA- haplotype to different extents. Donation of BM or PB depended on donor willingness. The HLA-A, HLA-B, and HLA-DRB1 typing results for the recipients were submitted to cord blood banks in P.R. China to identify compatible UCB units. The selection criteria for UCB were based on the results of HLA typing (requiring at least 3 out of 6 matched HLA loci between the UCB and recipient), cell doses evaluated before freezing (ensuring a minimum of 1 × 10^4^ CD34^+^ cells per kilogram of recipient weight), and blood type (accounting same blood type between UCB and recipient/donor).

A myeloablative, Bu/Cy-based regimen with ATG (Sanofi Genzyme, Cambridge MA, USA) was used for all patients within five days after randomization. The conditioning protocol comprised the following: Me-CCNU 250 mg/m^2^ (day −10), Ara-C 2 g/m^2^ (days −9 and −8, every 12 h), busulfan 4 mg/kg/d (days −7 to day −5), cyclophosphamide 1.8 g/m^2^/d (days −4 and −3), and ATG 2.5 mg/kg/d (days −5 to −2). Eight hours prior to the infusion of the haploidentical graft, The UCB unit was infused right after resuscitation. Fresh and unmanipulated BM or PB mononuclear cells were infused on the same day they were collected. GVHD prophylaxis containing cyclosporin A (from −10 day with a target blood concentration ranging of 200–300 ng/mL), methotrexate (day +1: 15 mg/m^2^, day +3, day +6 and day +11: 10 mg/m^2^) and mycophenolate mofetil (days −10 to +30: 1 g oral intake twice a day, then gradually reduced the dosage until day +60) was used in all patients. Patients were given supportive care and posttransplantation surveillance following the procedures described earlier.^17,27^

After transplantation, PB is regularly monitored to assess MRD, chimerism, organ function, GVHD and infections (twice a week for the first month after enrollment, weekly from the 2nd to 3rd month after enrollment, once every two weeks from the 4th to 6th month after enrollment, monthly from the 7th to 24th month after enrollment, and thereafter, once every three months until the study is completed). BM assessment is performed to monitor MRD and chimerism (monthly during the first six months posttransplantation, every three months from the 6th to 24th month posttransplantation, and thereafter, every six months until the study is completed). Assessments of PB and BM were repeated when clinically indicated. Chimerism was monitored by multiparameter fluorescent short tandem repeats analyses and single nucleotide polymorphisms-based NGS (SNP-NGS).^49^ Remission status was evaluated based on FCM-based MRD (FCM-MRD), fusion gene and gene mutation in BM (detailed in Supplementary Materials and Methods). CMV viremia and EBV viremia were detected using qPCR. Immune cell subsets posttransplantation were detected using multicolor FCM (detailed in Supplementary Materials and Methods).

### Endpoints

The primary endpoint was 3-year OS, defined as the duration from randomization until death from any cause or until the last follow-up. Secondary endpoints included PFS, CIR, NRM, and AEs. PFS was measured as the probability of surviving without disease progression at a given point in time. NRM was defined as death without disease progression attributed to the transplantation process. Relapse was defined as the presence of at least 5% blasts in BM, the reappearance of blasts in PB, or the development of extramedullary disease. AEs were recorded within 2 years posttransplantation and graded using the National Cancer Institute Common Terminology Criteria for Adverse Events (CTCAE v4) with the exception of GVHD. The assessment of aGVHD and cGVHD as AEs were evaluated in accordance with published reports (detailed in Supplementary Clinical Study Protocol).^50,51^ The occurrence of relapse or death resulting from relapse was not documented as an adverse event.

### Statistical analysis

The aim of this trial was to investigate the hypothesis that coinfusion of UCB would improve OS in AML patients with haplo-HCT. Preliminary findings for 51 haplo-cord HCT and 87 haplo-HCT AML patients treated at the First Affiliated Hospital of Soochow University between 2011–2013 showed 77.3% and 60.1% 3-year OS, respectively. Based on the retrospective results, the sample size calculation was performed using PASS software (version 15.0), revealing that a minimum of 134 patients in each group, accounting for a drop-out rate of 6.0%, would be required to achieve a significance level of 0.05 and a power of 90%.

Efficacy endpoints in this study were analyzed in the ITT population, which consisted of randomly assigned patients. Safety analysis was performed in patients who received family donors with/without UCB graft infusion. Comparisons of categorical variables were assessed using either the chi-squared test or Fisher’s exact test. Comparisons of continuous variables were performed using either two-sample t-tests or Mann‒Whitney U tests. CIR and NRM were considered and estimated by competing risks: NRM was considered a competing risk for CIR, and relapse was considered a competing risk for NRM. Gray’s test and the Fine and Gray model were used to analyze cumulative incidences. Analyses of OS and PFS were conducted utilizing the Kaplan‒Meier method and assessed with the log-rank test. The HR and its 95% CI were estimated via the Cox proportional hazards model to determine their corresponding values.

Subgroup post- hoc analysis was conducted on OS comparing the haplo-cord HCT and haplo-HCT groups. Univariate and multivariate analyses were performed in the ITT population. Post- hoc analysis of cumulative incidences of neutrophil and platelet engraftment, 100-day NRM, grade II-IV aGVHD, moderate/severe cGVHD, infection (more than CTCAE grade 3), CMV viremia, EBV viremia and HC were performed in the patients who received graft infusion. MRD-response at one month, 100 days and six months posttransplantation, aGVHD-involved organs and chimerism were also post- hoc analyzed in the patients who received graft infusion. Graft chimerism and immune reconstitution of T cells, B cells and NK cells were post- hoc exploratorily analyzed. Definitions of neutrophil and platelet engraftment, CMV and EBV viremia, MRD-response, complete engraftment and immune reconstitution were detailed in Supplementary Materials and Methods. Time-to-event risk factors were analyzed by the time-dependent Cox regression model when the test indicated the validity of proportional hazard assumptions. Variables of sex, age, 2022 ELN adverse risk,^52^ white blood cell count at diagnosis, inducting chemotherapy courses, extramedullary leukemia, disease status at transplantation, coinfusion of UCB, infection, grade II-IV aGVHD, moderate/severe cGVHD and center were included in univariable analysis. In multivariate analyses, only covariates with a p-value < 0.1 from univariate analyses were applied. All tests were statistically different with a cutoff level of 0.05 (two-tailed) except for the superiority hypothesis. SPSS (version 26.0) and R (version 4.0.2) were used for data analyses.

### Supplementary information

Supplementary Materials

## Data Availability

Data can be reasonably requested via E-mail (proposals should be directed to yangxu@suda.edu.cn). Participants’ data devoid of names or identifying information will be made accessible upon approval from all corresponding authors. The study protocol is available in the Supplementary Materials and Methods.
