# Supplementary material for: Haploidentical hematopoietic cell transplantation with or without an unrelated cord blood unit for adult acute myeloid leukemia: a multicenter, randomized, open-label, phase 3 trial
Source: Signal Transduct Target Ther. 2024 May 6;9:108. doi: 10.1038/s41392-024-01820-5 (PMC11070414; doi:10.1038/s41392-024-01820-5)
Supplement: Supplementary file 1 — Supplementary Materials [file 41392_2024_1820_MOESM1_ESM.docx]

Supplementary Materials for

Haploidentical hematopoietic cell transplantation with or without an unrelated cord blood unit for adult acute myeloid leukemia: a multicenter, randomized, open-label, phase 3 trial

Biqi Zhou, Jia Chen, Tianhui Liu, Yishan Ye, Yanming Zhang, Yiyang Ding, Hong Liu, MingQing Zhu, Xiao Ma, Xiaoli Li, Longfei Zhao, Zhihong Lin, He Huang, Yang Xu, and Depei Wu

Correspondence to: [drwudepei@163.com](mailto:drwudepei@163.com), [yangxu@suda.edu.cn](mailto:yangxu@suda.edu.cn), [huanghe@zju.edu.cn](mailto:huanghe@zju.edu.cn) and chenjia@suda.edu.cn

**This PDF file includes:**

Materials and Methods

Figures S1 to S6

Tables S1 to S5

Clinical Study Protocol

**Materials and Methods**

Remission status evaluation

During follow-up, a panel of 10 markers based on the patient’s leukemia-associated phenotypes at diagnosis, including CD38, CD13, CD33, CD117, CD34, CD10, HLA-DR, CD45, CD19, and one marker from among CD7, CD20 or CD123, was used to analyze flow cytometry-based measurable residual disease (FCM-MRD). FCM-MRD was defined as cells with abnormal expression patterns, changes in expression intensity and aberrant expression and was quantified as a percentage of total CD45^+^ cells. Quantitative polymerase chain reaction (qPCR) analysis based on standard plasmids constructed in our laboratories was performed to measure copies of fusion genes detected at diagnosis. More than 1×10^-4^ fusion gene copies were defined as positivity. Gene mutations detected at diagnosis were detected in genomic DNA by Sanger sequencing.

Immune cell subsets detecting

Multiparameter flow cytometry was performed to investigate the immune cell subsets reconstitution at two-weeks, one-month, two-months, 100-days, six-months, nine-months, 12-months, 18-months and 24-months posttransplantation. T cells, NK cells and B cells were defined as CD3^+^, CD3^-^CD56^+^ and CD3^-^CD19^+^ populations, respectively. CD3^+^CD4^+^ and CD3^+^CD8^+^ and CD3^+^CD4^-^CD8^-^ TCR_γ/δ_^+^ populations were considered as CD4^+^, CD8^+^ and γδT cells. The markers used to differentiate T-cell subsets were as follows: T_naive_ cells: CD3^+^CCR7^+^CD45RA^+^; T_em_ cells: CD3^+^CCR7^-^CD45RA^-^; T_cm_ cells: CD3^+^CD4^+^CCR7^+^CD45RA^-^; T_reg_ cells: CD3^+^CD4^+^CD25^+^ CD127^±^; T_em_-RA cells: CD3^+^CD8^+^CCR7^-^CD45RA^+^; Th1 cells: CD3^+^CD4^+^CXCR3^+^ CCR6^-^; Th2 cells: CD3^+^CD4^+^CXCR3^-^CCR6^-^; Th17 cells: CD3^+^CD4^+^CXCR3^-^CCR6^+^; Tc1 cells: CD3^+^CD8^+^CXCR3^+^CCR6^-^; Tc2 cells: CD3^+^CD8^+^CXCR3^-^CCR6^-^; Tc17 cells: CD3^+^CD8^+^CXCR3^-^CCR6^+^. The antibodies used for flow cytometry are listed as follows: DURAClone (C03216, Beckman Coulter), DURAClone (C03217, Beckman Coulter), PerCP5.5 anti-human CD3 (clone HIT3a, BioLegend), APC/CY7 anti-human CD4 (clone A161A1, BioLegend), PE/CY7 anti-human CD8 (clone SK1, BioLegend), FITC anti-human TCR γ/δ (clone B1, BioLegend), BV605 anti-human CD183 (CXCR3) (clone G025H7, BioLegend), BV421 anti-human CD196 (CCR6) (clone G034E3, BioLegend), APC anti-human Ki67 (clone Ki-67, BioLegend). The acquisition was performed on Navios EX (Beckman Coulter, Brea, USA) or FACS NovoCyte (ACEA Biosciences, San Diego, USA) and live cells were gated based on FSC-A and SSC-A characteristics. Data were analyzed using FlowJo v10 software.

Definitions

Neutrophil engraftment was defined as the first of three consecutive days when the neutrophil count reached >0.5×10^9^/L. Platelet engraftment was defined as the first of seven consecutive days when the platelet count reached >20×10^9^/L without transfusion support. CMV and EBV viremia were defined as >10^2^ copies/mL DNA load. MRD-response was defined as FCM-MRD <1.00×10^-3^, a 3-log reduction from the fusion gene transcript level at diagnosis and conversion of gene mutation from positive to negative in BM. Complete engraftment was defined as the chimerism of the dominant graft maintained at >95% in BM for more than 100 days posttransplantation. Immune reconstitution was defined as cell counts similar to the normal range for healthy adults.


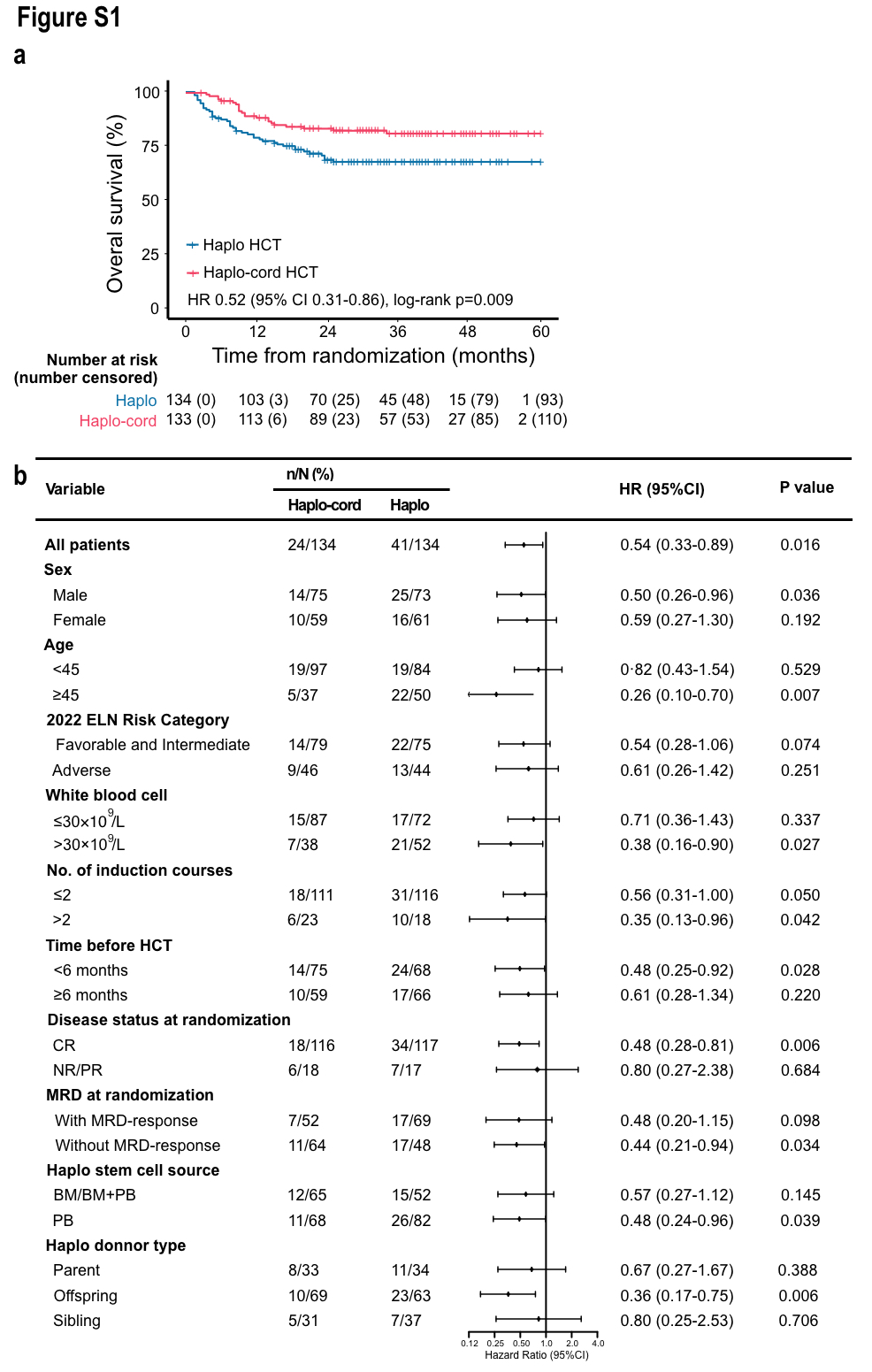


**Supplementary Figure S1.**

**a** Overall survival (OS) in patients who received family donors with/without UCB graft infusion. **b** Subgroup analysis of 3-year OS in haplo-cord HCT and haplo-HCT patients in ITT population. PR=partial response and NR=nonresponse.


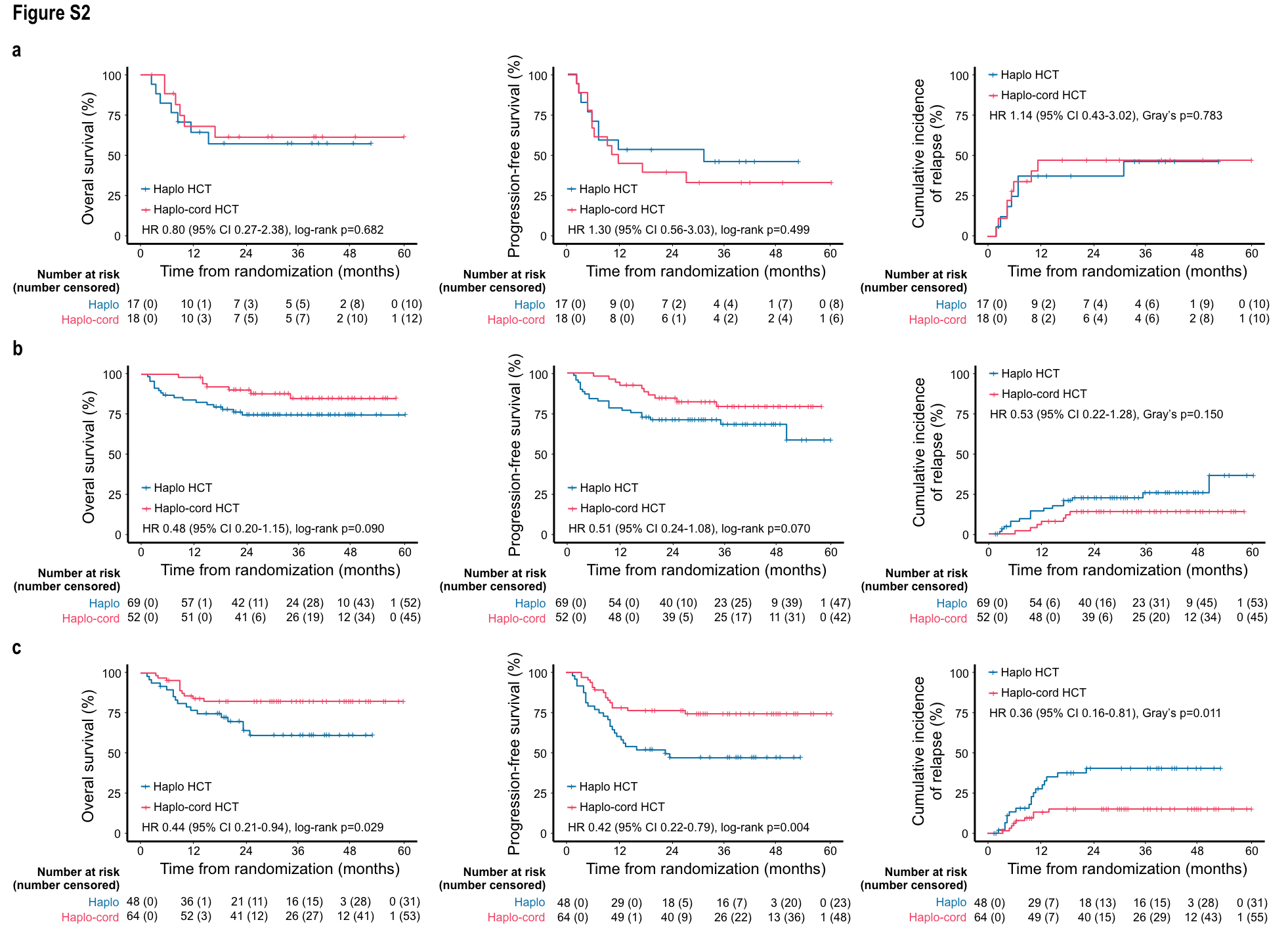


**Supplementary Figure S2.**

**a** Kaplan‒Meier plots of OS and progression-free survival (PFS), and cumulative incidence curves of the cumulative incidence of relapse (CIR) in patients who were partial response or nonresponse pre-HCT. **b** Kaplan‒Meier plots of OS and PFS, and cumulative incidence curves of the CIR in patients who achieved CR with MRD-response pre-HCT. **c** Kaplan‒Meier plots of OS and PFS, and cumulative incidence curves of the CIR in patients who achieved CR without MRD-response pre-HCT.


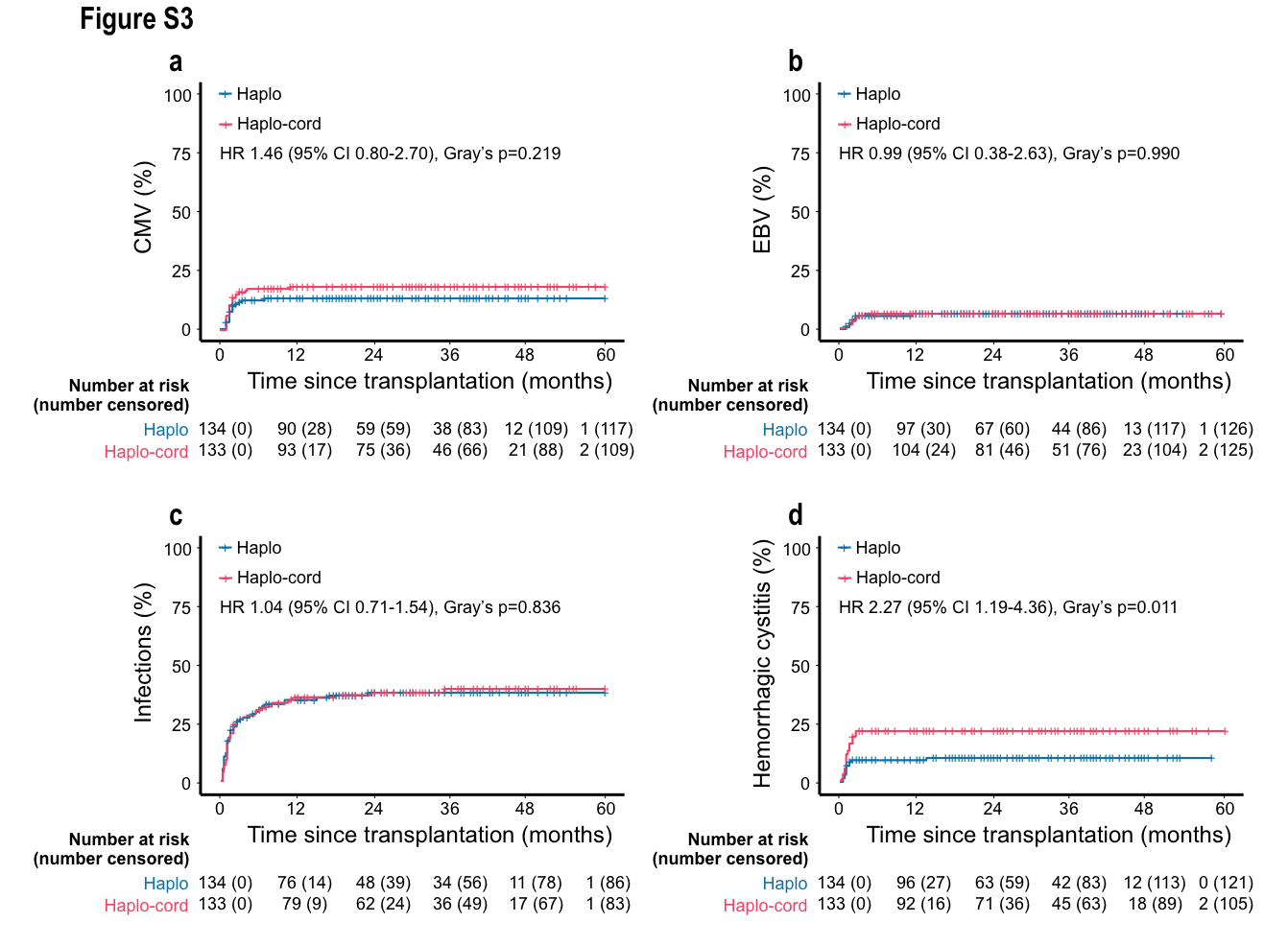


**Supplementary Figure S3.**

**a** Cumulative incidence curves of CMV viremia infection. **b** Cumulative incidence curves of EBV viremia infection. **c** Cumulative incidence curves of infections. **d** Cumulative incidence curves of [hemorrhagic cystitis](javascript:;).


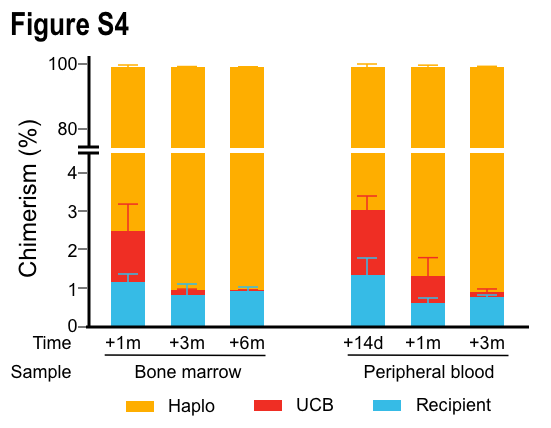


**Supplementary Figure S4.**

Chimerism of recipient, haploidentical donor and unrelated cord blood unit in the haplo-cord HCT cohort detected by single nucleotide polymorphisms-based next-generation sequencing. Data are presented as mean ± SEM.


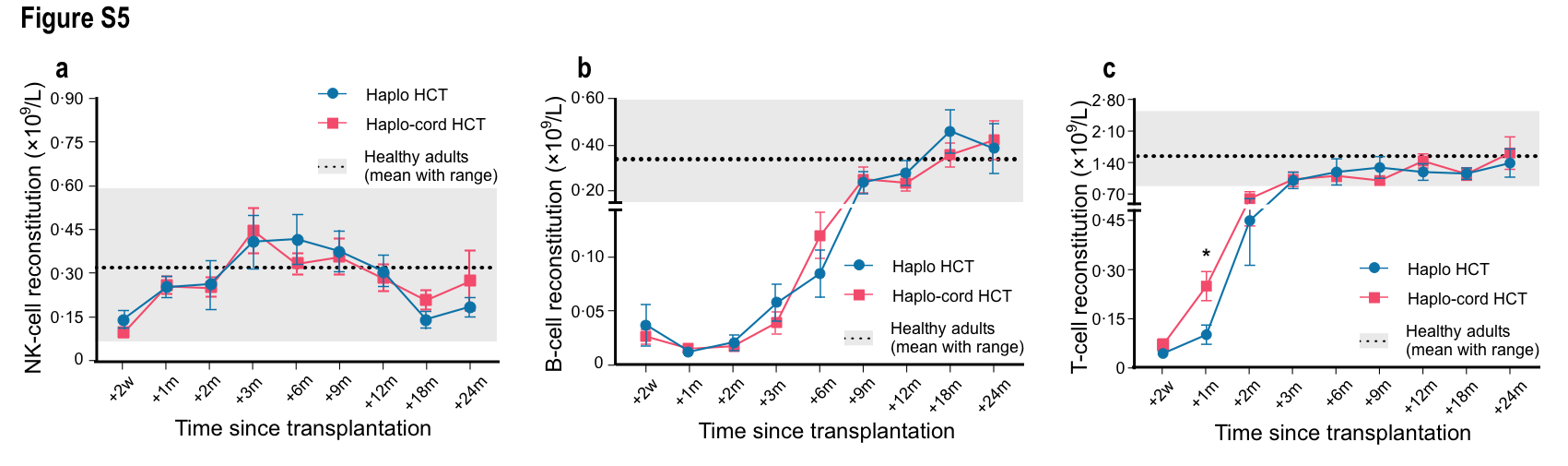


**Supplementary Figure S5.**

a NK-cell reconstitution in the haplo-cord HCT and haplo-HCT groups. b B-cell reconstitution in the haplo-cord HCT and haplo-HCT groups. c T-cell reconstitution in the haplo-cord HCT and haplo-HCT groups. Data are presented as mean ± SEM. *p<0.05.


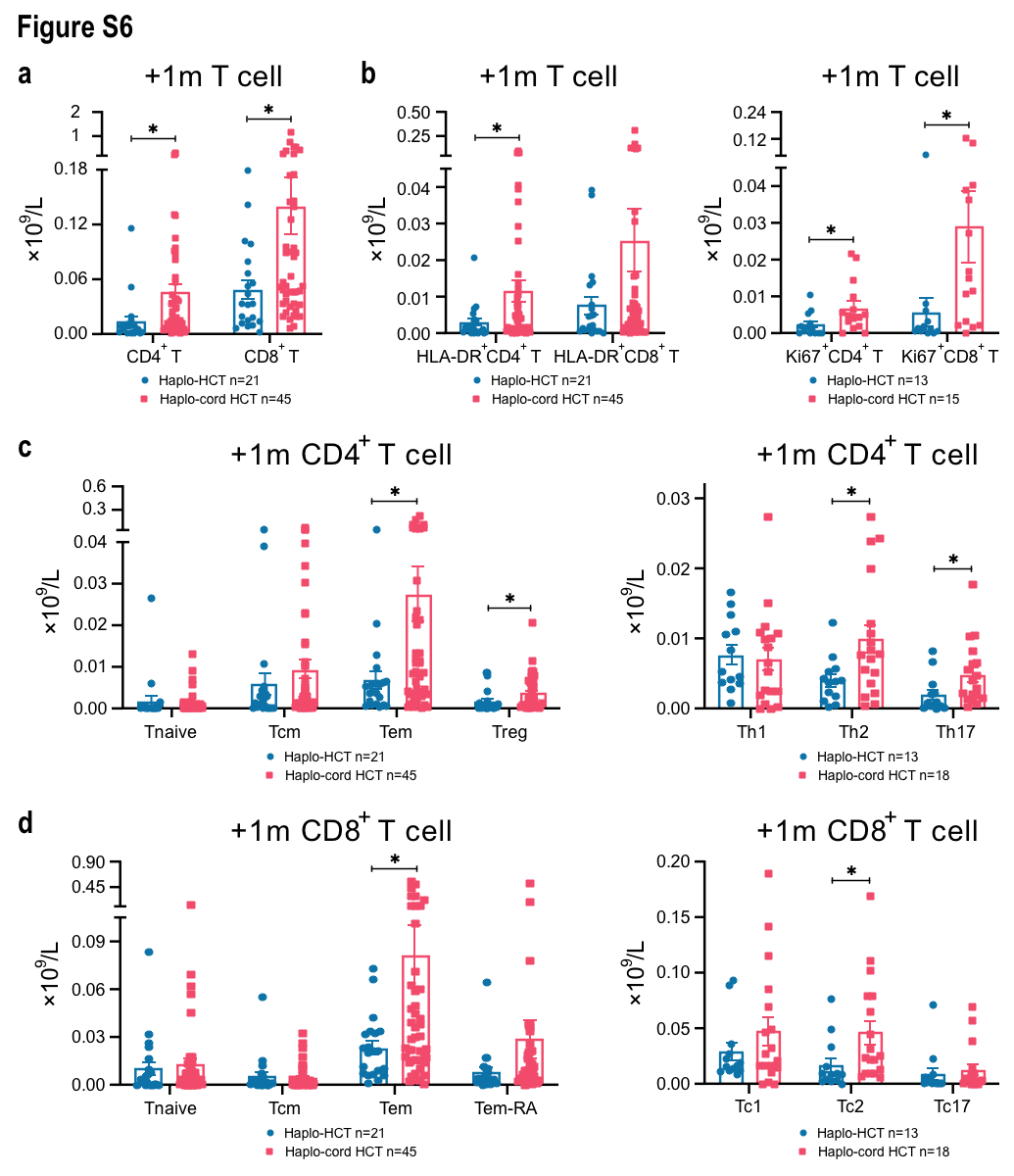


**Supplementary Figure S6.**

**a** Absolute counts of CD4+ T cells, CD8+ T cells and γδT cells, **b** Absolute counts of HLA-DR expressed (left) and proliferative (right) CD4+ T cells and CD8+ T cells, **c-d** Differentiated T-cell subsets in the haplo-cord HCT and haplo-HCT recipients. Data are presented as mean ± SEM. *p<0.05.

**Table S1. List of participating study centers**

| **Study site** | **Principal investigator** |
| --- | --- |
| The First Affiliated Hospital of Soochow University | Dr. Depei Wu |
| The First Affiliated Hospital, Zhejiang University School of Medicine | Dr. He Huang |
| The Affiliated Huai'an Hospital of Xuzhou Medical University, and the Second People's Hospital of Huai'an | Dr. Yanming Zhang |
| The Soochow Hopes Hematology Hospital | Dr. Xiaoli Li |
| The Hygeia Suzhou Yongding Hospital | Dr. Zhihong Lin |

**Table S2. Causes of death in the intention-to-treat (ITT) population**

| **Group** | **Haplo-cord HCT** | **Haplo-HCT** | **P** |
| --- | --- | --- | --- |
|  | **N=24** | **N=41** |  |
| Relapse | 11 (45.83) | 18 (43.90) | 0.998 |
| graft-versus-host disease | 4 (16.67) | 8 (19.51) |  |
| Severe infection | 5 (20.83) | 9 (21.95) |  |
| Bleeding | 1 (4.17) | 2 (4.88) |  |
| Others | 1 (4.17) | 1 (2.44) |  |
| Uncertain | 2 (8.33) | 3 (7.32) |  |

Data are the n (%).

**Table S3. Acute graft-versus-host disease (aGVHD)-involved organs**

|  | | **Haplo-cord HCT** | **Haplo-HCT** | **P** |
| --- | --- | --- | --- | --- |
| **Skin** | Total | 35 (26.3) | 23 (17.2) | 0.070 |
|  | Stage 1-2 | 25 (71.4) | 16 (69.6) | 0.155 |
|  | Stage 3 | 6 (17.1) | 7 (30.4) |  |
|  | Stage 4 | 4 (11.4) | 0 |  |
| **Intestines** | Total | 21 (15.8) | 13 (9.7) | 0.136 |
|  | Stage 1-2 | 16 (76.2) | 4 (30.8) | 0.015 |
|  | Stage 3 | 2 (9.5) | 1 (7.7) |  |
|  | Stage 4 | 3 (14.3) | 8 (61.5) |  |
| **Liver** | Total | 1 (0.8) | 3 (2.2) | 0.622 |
|  | Stage 1-2 | 0 | 3 (100.0) | 0.250 |
|  | Stage 3 | 1 (100.0) | 0 |  |
|  | Stage 4 | 0 | 0 |  |

Data are the n (%).

**Table S4. Baseline characteristics of complete UCB and mixed cord blood chimerism patients**

| **Patient ID** | **TG39** | **TG54** | **TG55** | **TG82** | **TG83** | **TG85** | **TG99** | **TG110** |
| --- | --- | --- | --- | --- | --- | --- | --- | --- |
| Sex | Female | Male | Female | Female | Male | Male | Female | Female |
| Age, year | 37 | 33 | 40 | 36 | 54 | 55 | 45 | 40 |
| Time before HCT, month | 4.50 | 4.00 | 6.50 | 6.00 | 12.50 | 5.50 | 5.50 | 8.00 |
| Disease status at transplantation | CR1 | CR1 | CR1 | CR1 | Relapse | CR1 | CR1 | CR1 |
| Chimerism | UCB | mixed | mixed | mixed | mixed | mixed | mixed | mixed |
| Haplo HLA | 5/10 | 5/10 | 5/10 | 5/10 | 5/10 | 5/10 | 5/10 | 6/10 |
| Haplo donor type | Offspring | Parent | Offspring | Offspring | Offspring | Offspring | Offspring | Offspring |
| Haplo mononuclear cells, ×10^8^/kg | 7.79 | 8.26 | 4.98 | 7.80 | 8.05 | 10.60 | 6.21 | 6.32 |
| Haplo CD34^+^ cells, ×10^6^/kg | 3.04 | 3.63 | 2.99 | 2.96 | 3.02 | 4.15 | 2.17 | 3.01 |
| UCB HLA | 4/6 | 5/6 | 5/6 | 4/6 | 5/6 | 6/6 | 5/6 | 4/6 |
| UCB mononuclear cells, ×10^6^/kg | 11.33 | 26.28 | 25.50 | 20.38 | 16.48 | 16.54 | 20.52 | 22.79 |
| UCB CD34^+^ cells, ×10^4^/kg | 9.76 | 7.60 | 1.66 | 6.10 | 7.90 | 2.98 | 4.31 | 9.80 |
| Neutrophil engraftment, day | 19 | 10 | 10 | 11 | 13 | 11 | 17 | 12 |
| Platelet engraftment, day | 21 | 11 | 11 | 12 | 24 | 11 | 96 | 13 |
| II-IV aGVHD, month | - | - | - | - | 0.75 | - | - | - |
| cGVHD, month | - | - | - | - | - | - | 6.50 | - |
| Severe infection, month | - | - | - | 1.50; 3.50 | - | - | - | 1 |
| CMV/EBV infection, month | - | - | - | - | - | - | - | 1 |
| Veno-occlusive disease, month | - | - | - | - | - | - | - | - |
| Relapse, month | - | - | - | - | 9.50 | - | - | - |
| Follow-up time, month | 18.00 | 16.00 | 6.00 | 42.00 | 39.50 | 55.00 | 44.50 | 5.50 |
| Prognosis | alive | alive | alive | alive | alive | alive | alive | died of infection |

HCT=hematopoietic cell transplantation, HLA=human leukocyte antigen, UCB=unrelated cord blood, cGVHD=chronic graft-versus-host disease, CMV=cytomegalovirus, EBV=Epstein‒Barr virus.

**Table S5. Immune reconstitution**

| **Cell type** | **Time** | **Haplo-cord HCT** | | | **Haplo-HCT** | | | **P** |
| --- | --- | --- | --- | --- | --- | --- | --- | --- |
|  |  | **median** | **IQR** | | **median** | **IQR** | |  |
| **NK cells** | +14 days | 0.07 | 0.03 | 0.10 | 0.09 | 0.06 | 0.16 | 0.213 |
|  | +1 month | 0.22 | 0.13 | 0.31 | 0.21 | 0.14 | 0.33 | 0.903 |
|  | +100 days | 0.38 | 0.17 | 0.52 | 0.27 | 0.18 | 0.56 | 0.756 |
|  | +6 months | 0.29 | 0.18 | 0.48 | 0.39 | 0.22 | 0.51 | 0.308 |
|  | +9 months | 0.26 | 0.14 | 0.47 | 0.43 | 0.19 | 0.49 | 0.867 |
|  | +12 months | 0.20 | 0.12 | 0.35 | 0.31 | 0.31 | 0.39 | 0.787 |
| **B cells** | +14 days | 0.01 | 0.00 | 0.03 | 0.01 | 0.00 | 0.02 | 0.520 |
|  | +1 month | 0.01 | 0.00 | 0.02 | 0.01 | 0.00 | 0.02 | 0.490 |
|  | +100 days | 0.01 | 0.01 | 0.03 | 0.02 | 0.01 | 0.11 | 0.348 |
|  | +6 months | 0.08 | 0.01 | 0.19 | 0.08 | 0.01 | 0.11 | 0.373 |
|  | +9 months | 0.23 | 0.07 | 0.32 | 0.23 | 0.18 | 0.29 | 0.871 |
|  | +12 months | 0.21 | 0.10 | 0.33 | 0.24 | 0.19 | 0.39 | 0.480 |
| **T cells** | +14 days | 0.02 | 0.01 | 0.08 | 0.02 | 0.01 | 0.05 | 0.389 |
|  | +1 month | 0.13 | 0.08 | 0.33 | 0.06 | 0.03 | 0.09 | 0.034 |
|  | +100 days | 0.82 | 0.58 | 1.01 | 0.74 | 0.53 | 1.60 | 0.928 |
|  | +6 months | 0.83 | 0.65 | 1.29 | 0.75 | 0.55 | 1.59 | 0.754 |
|  | +9 months | 1.05 | 0.80 | 1.20 | 0.85 | 0.74 | 1.52 | 0.136 |
|  | +12 months | 1.33 | 0.71 | 1.87 | 1.10 | 0.61 | 1.46 | 0.339 |

The normal range of cell counts in our laboratory: NK cells: 0.07-0.59×10^9^/L, B cells: 0.15-0.60×10^9^/L, T cells: 0.90-0.56×10^9^/L.

# Clinical study protocol

**Protocol Title: An open-label, multicenter, randomized, phase 3 trial to assess the efficacy and safety of coinfusion with unrelated cord blood unit for patients with acute myeloid leukemia undergoing haploidentical hematopoietic cell transplantation**

**Sponsor: The First Affiliated Hospital of Soochow University**

**Version: 2.0**

**Date: 20170628**

# INVESTIGATOR'S STATEMENT

I have received and completely reviewed the following protocol (Protocol Version 3.0, dated 28 June 2017), including all appendices:

As Principal Investigator, I understand and agree to conduct this clinical study as described and will comply with the ethical and regulatory considerations delineated herein.

**Study Title**

An open-label, multicenter, randomized, phase 3 trial to assess the efficacy and safety of coinfusion with unrelated cord blood unit for patients with acute myeloid leukemia undergoing haploidentical hematopoietic cell transplantation

**Principal Investigator Signature and Contact Information**

| **Principal Investigator (print)** |  |
| --- | --- |
| **Principal Investigator (signature)** |  |
| **Date of Signature** |  |
| **Institution/Affiliation** |  |
| **City, Province, Country** |  |

# Study Synopsis

| **Study title** | An open-label, multicenter, randomized, phase 3 trial to assess the efficacy and safety of coinfusion with unrelated cord blood (UCB) unit for patients with acute myeloid leukemia (AML) undergoing haploidentical hematopoietic cell transplantation (haplo-HCT) |
| --- | --- |
| **Indication** | AML undergoing haplo-HCT |
| **Study phase** | 3 |
| **Study applicant** | The First Affiliated Hospital of Soochow University |
| **Study centers** | 5 study centers (the First Affiliated Hospital of Soochow University; The First Affiliated Hospital, Zhejiang University School of Medicine; the Affiliated Huai'an Hospital of Xuzhou Medical University, and the Second People's Hospital of Huai'an; the Soochow Hopes Hematology Hospital and the Soochow Yongding Hospital) |
| **Number of subjects planned** | Approximately 268 subjects (134 in each group) will be randomized. |
| **Study duration** | Estimated to be 6 years |
| **Objectives** | **Primary objective:**  To compare the 3-year overall survival (OS) in patients with AML undergoing haplo-HCT combined with UCB unit (haplo-cord HCT) versus haplo-HCT.  **Secondary objectives:**  To compare the progression-free survival (PFS), cumulative incidence of relapse (CIR), nonrelapse mortality (NRM), and adverse events (AEs) in patients with AML undergoing haplo-HCT combined with UCB unit (haplo-cord HCT) versus haplo-HCT. |
| **Study design** | This is an open-label, multicenter, randomized, phase 3 trial comparing coinfusion with UCB unit for AML patients undergoing haplo-HCT. Approximately 268 subjects will be randomized in a 1:1 ratio to receive haplo-cord HCT (134 subjects) or haplo-HCT (134 subjects). Randomization is performed with permuted blocks (block size four) and implemented through an interactive web-based response system.  **Haplo-cord HCT Group:** Coinfusion of unrelated UCB unit with haploidentical graft.  **Haplo-HCT Group:** Haploidentical graft infusion alone (without UCB unit infusion).  Disease assessment including routine peripheral blood (PB) and bone marrow (BM) assessment will be regularly performed posttransplantation. Routine blood is monitored before randomization, twice a week for the first month after enrolment, once a week from the 2nd to 3rd month after enrolment, once every two weeks from the 4th to 6th month after enrolment, once every month from the 7th to 24th month, and then once every three months until the study is completed. BM assessment is performed before randomization, every month for the first six months posttransplantation, every three months from the 6th to 24th month posttransplantation, and then every six months until the study is completed. Assessment of PB and BM should be repeated when clinically indicated.  Safety and tolerability will be assessed in all subjects within 2 years posttransplantation. Except for graft-versus-host disease (GVHD), all AEs are graded according to CTCAE version 4.0. Acute GVHD (aGVHD) and chronic GVHD (cGVHD) are graded as AEs according to our self-defined criteria. |
| **Inclusion criteria** | Subjects eligible for enrolment in this study must meet all of the following criteria:  1. Age 18 to 60 years old  2. Patients with AML  3. With available minimal residual disease (MRD) parameters assessed by flow cytometry (FCM) and/or quantitative polymerase chain reaction (qPCR)  4. Having no available HLA-matched donor, willing to undergo haplo-HCT and having a suitable haploidentical donor  5. With Eastern Cooperative Oncology Group (ECOG) performance status of 0-3  6. Signing an informed consent form, having the ability to comply with study and follow-up procedures |
| **Exclusion criteria** | Subjects meeting any of the following criteria are ineligible for this study:  1. Acute promyelocytic leukemia (AML subtype M3)  2. With other malignancies  3. Failing to acquire a suitable UCB unit  4. With a previous history of autologous hematopoietic cell transplantation (auto-HCT), allogeneic hematopoietic cell transplantation (allo-HCT) or chimeric antigen receptor T-cell therapy  5. With uncontrolled infection intolerant to haplo-HCT  6. With severe organ dysfunction  ▪ Cardiac dysfunction (particularly congestive heart failure, unstable coronary artery disease and serious cardiac ventricular arrhythmias requiring antiarrhythmic therapy)  ▪ Respiratory failure (PaO2≤60 mmHg)  ▪ Hepatic abnormalities (total bilirubin≥2×upper limit of normal [ULN], alanine aminotransferase or aspartate aminotransferase≥2×ULN)  ▪ Renal dysfunction (creatinine≥2 mg/dL creatinine clearance rate < 30 mL/min)  7. In pregnancy or lactation period  8. With any conditions not suitable for the trial (investigators' decision) |
| **Study treatment** | **Haplo-cord HCT Group:**  For subjects randomized to haplo-cord HCT group, all [participant](javascript:;)s received Bu/Cy-based regimen. The UCB unit is infused 8 hours before the haploidentical graft infusion.  **Haplo-HCT Group:**  For subjects randomized to haplo-HCT group (control group), all [participant](javascript:;)s received Bu/Cy-based regimen. No UCB unit is infused. |
| **Sample size determination** | This trial is designed to test the hypothesis that coinfusion with UCB would improve OS in AML patients with haplo-HCT. The sample size calculation is based on the primary endpoint, the 3-year OS, which was 77.3% and 60.1% in haplo-cord HCT and haplo-HCT patients with AML among retrospective results in the First Affiliated Hospital of Soochow University between 2011-2013. A minimum of 268 patients (134 in each group) including a drop-out rate of 6.0% is required to provide the study with a significance level of 0.05 and a power of 90%. |
| **Statistical analysis** | Efficacy analysis is performed based on the intention-to-treat (ITT) population, which includes all randomized subjects who received graft infusion. The safety population included patients who received graft infusion.  **Primary Efficacy Analysis:**  The primary endpoint of this study is 3-year OS, which is defined as the time from randomization until death from any cause or last follow-up. OS is estimated using Kaplan‒Meier method and log-rank test.  **Secondary Efficacy Analysis:**  The secondary endpoints of this study are PFS, CIR and NRM. PFS is estimated using Kaplan‒Meier method and log-rank test. CIR and NRM are calculated by accounting for competing risks: relapse is a competing risk for NRM, and NRM is a competing risk for CIR. The comparison of CIR, NRM and engraftment between the two groups is tested using Gray’s test and Fine and Gray model.  ▪ PFS is defined as the time from randomization until disease progression or relapse or death from any cause.  ▪ NRM is defined as death from any cause not subsequent to relapse or disease progression.  ▪ Relapse is defined as either at least 5% of blasts in bone marrow (BM), the reappearance of blasts in PB or the development of extramedullary disease.  **Safety Analysis:**  Safety and tolerability will be assessed by incidence and severity of AEs and changes from baseline of all relevant parameters, including laboratory test values, physical examination, vital signs, and ECOG performance scores. Except for GVHD, all AEs are graded according to CTCAE version 4.0. aGVHD and cGVHD are graded as AEs according to our self-defined criteria. All subjects will be monitored for AEs within 2 years posttransplantation. |

# Table of contents

PAGE

[Clinical study protocol 13](#_Toc135911591)

[Investigator’s statement 14](#_Toc135911592)

[Study Synopsis 15](#_Toc135911593)

[Table of contents 20](#_Toc135911594)

[Abbreviations 22](#_Toc135911595)

[1. Introduction 24](#_Toc135911596)

[2. Study objectives 25](#_Toc135911597)

[2.1 Primary objective 25](#_Toc135911598)

[2.2 Secondary objectives 25](#_Toc135911599)

[3. Study design 25](#_Toc135911600)

[4. Subject selection criteria 29](#_Toc135911601)

[4.1 Subject selection criteria 29](#_Toc135911602)

[4.1.1 Number of subjects 29](#_Toc135911603)

[4.1.2 Inclusion criteria 29](#_Toc135911604)

[4.1.3 Exclusion criteria 29](#_Toc135911605)

[4.2 Withdrawal criteria 30](#_Toc135911606)

[5 Study procedures 30](#_Toc135911607)

[5.1 Screening 30](#_Toc135911608)

[5.2 Treatment allocation and blinding 30](#_Toc135911609)

[5.3 Study treatment 31](#_Toc135911610)

[5.3.1 Haplo-cord HCT group 31](#_Toc135911611)

[5.3.2 Haplo-HCT group 31](#_Toc135911612)

[5.4 Follow-up 31](#_Toc135911613)

[6. Efficacy Assessments 31](#_Toc135911614)

[6.1 Definitions 31](#_Toc135911615)

[6.2 Primary Efficacy Endpoint 32](#_Toc135911616)

[6.3 Secondary Efficacy Endpoint 32](#_Toc135911617)

[6.4 Schedule and Methods of Efficacy Assessments 32](#_Toc135911618)

[7. Safety Evaluation 32](#_Toc135911619)

[7.1 Medical History 33](#_Toc135911620)

[7.2 Vital Signs and Physical Examination 33](#_Toc135911621)

[7.3 Clinical Symptoms 33](#_Toc135911622)

[7.4 Clinical Laboratory Evaluations 33](#_Toc135911623)

[8. Adverse Events and Serious Adverse Events 34](#_Toc135911624)

[8.1 Definitions 34](#_Toc135911625)

[8.1.1 Adverse Events 34](#_Toc135911626)

[8.1.2 Serious Adverse Events 34](#_Toc135911627)

[8.2 Assessment of Severity 35](#_Toc135911628)

[8.3 Assessment of Causality 35](#_Toc135911629)

[8.4 Recording and Reporting Adverse Events and Serious Adverse Events 36](#_Toc135911630)

[9. Rules of Withdrawal 36](#_Toc135911631)

[9.1 Subjects Withdraw from the Study 36](#_Toc135911632)

[9.2 Premature Termination of the Study 36](#_Toc135911633)

[10.Rules of Follow-up 37](#_Toc135911634)

[10.1 Follow-up Period 37](#_Toc135911635)

[10.2 Visit Scheduling 37](#_Toc135911636)

[10.3 Contents 37](#_Toc135911637)

[11. Data Analysis and Statistical Considerations 37](#_Toc135911638)

[11.1 Hypotheses 37](#_Toc135911639)

[11.2 Study Design Considerations 37](#_Toc135911640)

[11.2.1 Sample Size Assumptions 38](#_Toc135911641)

[11.2.2 Primary Efficacy Endpoint 38](#_Toc135911642)

[11.2.3 Secondary Efficacy Endpoints 38](#_Toc135911643)

[11.3 Data Analysis Considerations 38](#_Toc135911644)

[11.3.1 Analysis Population 38](#_Toc135911645)

[11.3.2 Analysis Plan 38](#_Toc135911646)

[11.3.2.1 Baseline Data 38](#_Toc135911647)

[11.3.2.2 Analysis of Efficacy 39](#_Toc135911648)

[11.3.2.3 Analysis of Safety 39](#_Toc135911649)

[12. Materials for the Study 39](#_Toc135911650)

[13. Ethical Considerations 39](#_Toc135911651)

[13.1 Responsibility of Investigators 39](#_Toc135911652)

[13.2 Informed Consent Process 40](#_Toc135911653)

[13.3 Good Clinical Practice 40](#_Toc135911654)

[13.4 Protection of Subjects’ Personal Data 40](#_Toc135911655)

[14. Administrative Requirements 40](#_Toc135911656)

[15. References 41](#_Toc135911657)

[16. Appendices 43](#_Toc135911658)

[16.1 Monitoring Schema 43](#_Toc135911659)

[16.2 Appendix 1 Diagnosis and Classification of aGVHD and cGVHD 45](#_Toc135911660)

[16.3 Appendix 2 ECOG Performance Status 50](#_Toc135911661)

# Abbreviations

| AE | adverse event |
| --- | --- |
| ADL | activities of daily living |
| AEs | adverse events |
| aGVHD | acute graft-versus-host disease |
| allo-HCT | allogeneic hematopoietic cell transplantation |
| ALT | alanine aminotransferase |
| AML | acute myeloid leukemia |
| AST | aspartate aminotransferase |
| auto-HCT | autologous hematopoietic cell transplantation |
| BM | bone marrow |
| cGVHD | chronic graft-versus-host disease |
| CIR | cumulative incidence of relapse |
| CR | complete remission |
| CR1 | first complete remission |
| CRF | case report form |
| CTCAE | common Terminology Criteria for Adverse Events |
| DFS | disease-free survival |
| ECOG | Eastern Cooperative Oncology Group |
| FCM | flow cytometry |
| GCP | good Clinical Practice |
| G-CSF | granulocyte Colony-Stimulating Factor |
| GVHD | graft-versus-host disease |
| GVL | [graft-versus-tumor effect](javascript:;) |
| haplo-HCT | haploidentical hematopoietic cell transplantation |
| HR | hazard ratio |
| mITT | modified intent-to-treat |
| MRD | minimal residual disease |
| NIH | National Institutes of Health |
| NR | no evidence of response |
| NRM | nonrelapse mortality |
| OS | overall survival |
| PB | peripheral blood |
| PFS | progression-free survival |
| PR | partial remission |
| qPCR | quantitative polymerase chain reaction |
| SAE | serious adverse event |
| UCB | unrelated cord blood |
| ULN | the upper limit of normal |

# 1. Introduction

Acute myeloid leukemia (AML) is the most common leukemia in adults, accounting for nearly 80% of cases in this group.^1,2^ Although many patients with AML have a response to induction chemotherapy, relapse represents the major cause of treatment failure and results in unfavorable overall survival (OS).^3^ Several subtypes of AML, such as core-binding factor AML, could benefit from regular chemotherapy or autologous transplantation.^4^ Additionally, a growing body of studies has demonstrated that targeted drugs could prevent relapse. For example, sorafenib could prolong the remission duration of FLT3-ITD AML.^5-7^ For the majority of patients with AML, allogeneic hematopoietic cell transplantation (allo-HCT) remains the only potentially curative and effective treatment available. However, the prognosis is still unsatisfactory after transplantation. The incidence of relapses for AML patients who underwent allo-HCT in first complete remission (CR1) is approximately 30-40%, showing poor survival since most relapses occur in the first year after transplant.^8^ Haploidentical hematopoietic cell transplantation (haplo-HCT) usually tends to have a more powerful [graft-versus-tumor effect](javascript:;) (GVL) than HLA-matched HCT dose. However, haplo-HCT is also accompanied by an increased incidence of graft versus host disease (GVHD), which is the main cause of nonrelapse mortality (NRM).^1,9^ Umbilical cord blood allo-HCT has minimal risks for chronic GVHD (cGVHD) and powerful GVL^10^, while limited cell numbers result in a high risk of delayed engraftment.^11^

In the past few years, a transplantation strategy of a combination of haploidentical hematopoietic cells and unrelated cord blood (UCB) units (also known as haplo-cord HCT) has been proposed. Several reports have revealed some encouraging results both in myeloablative and non-myeloablative conditioning, which proved the feasibility of this approach.^12-16^ The Spanish group reported the data with a long-term follow-up, which demonstrated outcomes comparable to the outcome achieved with HLA-identical related donors in acute leukemia and myelodysplastic syndromes.^16^ Rapid engraftment, low GVHD and durable remissions were observed in the results with reduced-intensity conditioning by the study group of Dr van Besien.^17^ We previously summarized the outcomes of transplantation of unmanipulated haplo-HCT combined with the infusion of UCB in 50 patients with hematological malignancies, finding improved survival when compared with the historical data during 2003-2008.^12^ Similarly, improvement in OS and disease-free survival (DFS) were also found in high-risk leukemia group.^18^ These researches indicated that patients with hematological malignancies could benefit from haplo-cord HCT strategy. Currently, there are no reports about prospective randomized studies on haplo-cord HCT in the most common hematological malignancies, AML. Based on these, we designed this open-label, multicenter, randomized, phase 3 study to evaluate the efficacy and tolerability of coinfusion with UCB unit for AML patients undergoing haplo-HCT.

# 2. Study objectives

# 2.1 Primary objective

The primary objective of this study is to compare the 3-year OS in patients with AML undergoing haplo-HCT combined with UCB unit versus haplo-HCT.

# 2.2 Secondary objectives

The secondary objectives of this study are to compare the progression-free survival (PFS), cumulative incidence of relapse (CIR), NRM, and adverse events (AEs) in patients with AML undergoing haplo-HCT combined with UCB unit (haplo-cord HCT) versus haplo-HCT.

# 3. Study design

This is an open-label, multicenter, randomized, phase 3 trial comparing coinfusion with UCB unit for AML patients undergoing haplo-HCT. The study design is illustrated in Figure 1.

Subjects aged 18-60 with AML who are willing to undergo haplo-HCT will be screened for eligibility. Medical history evaluation, vital signs, physical examination, Eastern Cooperative Oncology Group (ECOG) performance status, peripheral blood (PB) and urine sampling for laboratory tests, electrocardiogram, chest imaging examination as well as bone marrow (BM) assessment will be performed to determine study eligibility, all of which should be performed no more than 7 days prior to randomization. Eligible subjects will be randomized in a 1:1 ratio to receive haplo-cord HCT or haplo-HCT. Randomization is performed with randomization codes generated by a computer-generated randomization system. Randomization should be performed no more than 5 days before conditioning.

Family donors should be ranked based on HLA match, age (younger preferred), gender (male preferred), and health status (better preferred). All donor-recipient pairs are typed at high resolution for HLA-A, HLA-B, HLA-C, HLA-DRB1 and HLA-DQB1 loci. All recipients receive grafts from family members sharing one HLA haplotype with the recipient but differed to a variable degree for the HLA antigens of the unshared HLA-haplotype. In addition to typing each donor-recipient pair, HLA typing is performed for parents and offspring and strictly analyzed to guarantee true haploid genetic background. Donation of bone marrow or peripheral blood depends on donor willingness. Granulocyte colony-stimulating factor (5 µg/kg/day for 5 days) is used to mobilize BM and PB. The target CD34^+^ count is more than 2×10^6^ per kilogram of recipient weight. Another collection of PBSCs is needed on day 02 or day 03 if cells collected on the previous days are insufficient. The fresh and unmanipulated BM or PB mononuclear cells are infused into the recipient on the day of their collection.

Recipients’ HLA-A, HLA-B, and HLA-DRB1 typing should be sent to cord blood banks in Shanghai, Beijing, or Shandong Province in China to look for ideal UCB units. The criteria for UCB selection are based on the results of HLA typing (UCB and recipient shared at least 3/6 matched HLA loci) and cell doses evaluated before freezing. HLA matching should be prioritized over cell dose. Suitable UCB units should have more than 1×10^4^ CD34^+^ cells per kilogram of recipient weight. At the same level of typing, the richest UCB unit is chosen. The blood type of UCB should also be considered, the same blood type between UCB and recipient or donor is preferred. All UCB units should be qualified clinical grade, normal in volume with depleted red blood cells, and transferred by cold-chain transportation. The UCB units should be infused into the recipient immediately after resuscitation.

Based on the randomization and assignment, the subjects will or will not receive coinfusion with a UCB unit after Bu/Cy-based conditioning regimen. Bu/Cy-based regimen consists of Me-CCNU 250 mg/m^2^ on day -10, Ara-C 2 g/m^2^ every 12 hours on days -9 and -8, busulfan 4 mg/kg/d on days -7 to -5, cyclophosphamide 1.8 g/m^2^/d on days -4 and -3, and rabbit anti-thymocyte globulin 2.5 mg/kg/d on days -5 to -2. The UCB unit should be infused eight hours before the haploidentical graft infusion (Figure 2). All subjects will receive cyclosporin A-based GVHD prophylaxis.^12^ Cyclosporin A is given starting on day -10 with a target blood concentration of 200 to 300 ng/mL; 15 mg/m^2^ methotrexate is given on day +1, and 10 mg/m^2^ methotrexate is given on day +3, day +6, and, in some cases, day +11. Oral mycophenolate mofetil 1.0 g twice daily is given from day -10 to day +30, then gradually tapered to day +60.

All subjects are isolated in sterile rooms from the beginning of the conditioning regimen. Infection prevention included selective gut decontamination (levofloxacin, albendazole, and fluconazole) before conditioning, and prophylactic antibiotics and antifungal therapies are administered during the conditioning immunosuppressive period.^12^ Ganciclovir 5mg/kg/q12h (days -9 to -2) is routinely used to prevent viral infection and eventually replaced by acyclovir to avoid marrow toxicity. Prophylactic IVIG is used once a week. Heparin and prostaglandin E1 are used to prevent sinusoidal obstruction syndrome. Granulocyte colony-stimulating factor (G-CSF) is administered subcutaneously to accelerate the recovery of neutrophils. Irradiated blood products are used to maintain a hemoglobin level above 60g/L and a platelet count over 20×10^9^/L.

Disease assessment including Routine blood and BM assessment will be regularly performed posttransplantation. Routine blood is monitored before randomization, twice a week for the first month after enrolment, once a week from the 2nd to 3rd month after enrolment, once every two weeks from the 4th to 6th month after enrolment, once every month from the 7th to 24th month, and then once every three months until the study is completed. BM assessment is performed before randomization, every month for the first six months posttransplantation, every three months from the 6th to 24th month posttransplantation, and then every six months until the study is completed. Assessment of PB and BM are repeated when clinically indicated.

FCM-MRD examination relies on the expression of aberrant surface antigens on AML blasts. A panel of 10 markers based on the patient’s leukemia-associated phenotypes at diagnosis, including CD38, CD13, CD33, CD117, CD34, CD10, HLA-DR, CD45, CD19, and one marker from among CD7, CD20 or CD123, is used to analyze FCM-MRD. Isotype-matched nonreactive mouse mAbs at the same protein concentrations should be used as negative controls. Analysis should be performed on a CD45 gate. Based on patients’ leukemia-associated phenotypes at diagnosis, FCM-MRD is defined as cells with abnormal expression patterns, change of expression intensity and aberrant expression. FCM-MRD is quantified as a percentage of total CD45^+^ cells. The cDNA synthesis of patients should be extracted according to the manufacturer’s protocols (Reverse Transcription Reagent Kit, Biological Materials Inc., BC, Canada). A quantitative polymerase chain reaction (qPCR) analysis based on standard plasmids constructed in laboratories should be performed to measure copies of fusion genes detected at diagnosis. The genomic DNA of patients should be extracted according to the manufacturer’s protocols (Genomic DNA mini-Kit, Invitrogen, Thermo Fisher Scientific, KS, US). Gene mutations detected at diagnosis should be detected by Sanger Sequencing. A qPCR analysis should be performed to measure copies of CMV and EBV.

Safety and tolerability will be followed within 2 years posttransplantation. Except for GVHD, all AEs are graded according to CTCAE version 4.0. Acute GVHD (aGVHD) and cGVHD are graded as AEs according to our self-defined criteria.


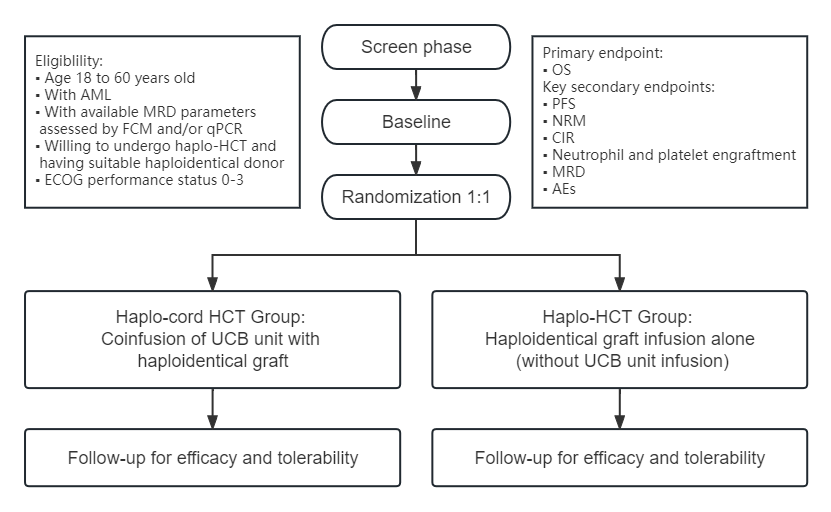


**Figure 1. Study** **Schema**

AML=acute myeloid leukemia; haplo-HCT=haploidentical hematopoietic cell transplantation; ECOG=Eastern Cooperative Oncology Group; OS=overall survival; PFS=progression-free survival; NRM=nonrelapse mortality; CIR=cumulative incidence of relapse; MRD=minimal residual disease; AEs= adverse effects.


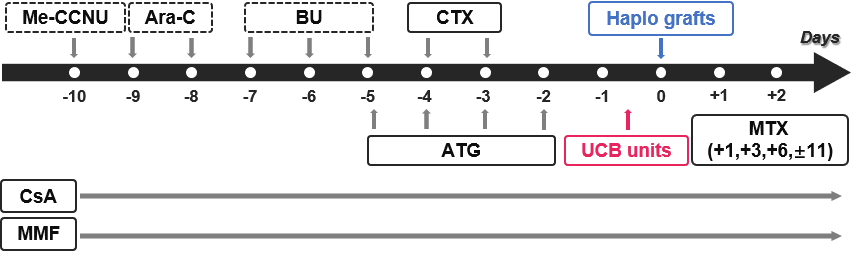


**Figure 2. Transplantation Schema**

BU=busulfan; CTX=cyclophosphamide; Haplo=haploidentical; ATG=rabbit antithymocyte globulin; UCB=unrelated cord blood; MTX=methotrexate; CsA=cyclosporin A; MMF=mycophenolate mofetil.

# 4. Subject selection criteria

# 4.1 Subject selection criteria

# 4.1.1 Number of subjects

Approximately 268 subjects will be randomized to haplo-cord HCT or haplo-HCT group (134 subjects per group).

# 4.1.2 Inclusion criteria

Subjects eligible for enrolment in this study must meet all of the following criteria:

1. Age 18 to 60 years old

2. Patients with AML

3. With available MRD parameters assessed by flow cytometry (FCM) and/or qPCR

4. Having no available HLA-matched donor, willing to undergo haplo-HCT and having a suitable haploidentical donor

5. With Eastern Cooperative Oncology Group (ECOG) performance status of 0-3

6. Signing an informed consent form, having the ability to comply with study and follow-up procedures

# 4.1.3 Exclusion criteria

Subjects meeting any of the following criteria are ineligible for this study:

1. Acute promyelocytic leukemia (AML subtype M3)

2. With other malignancies

3. Failing to acquire a suitable UCB unit

4. With a previous history of autologous hematopoietic cell transplantation (auto-HCT), allogeneic hematopoietic cell transplantation (allo-HCT) or chimeric antigen receptor T-cell therapy

5. With uncontrolled infection intolerant to haplo-HCT

6. With severe organ dysfunction

▪ Cardiac dysfunction (particularly congestive heart failure, unstable coronary artery disease and serious cardiac ventricular arrhythmias requiring antiarrhythmic therapy)

▪ Respiratory failure (PaO2≤60 mmHg)

▪ Hepatic abnormalities (total bilirubin≥2×upper limit of normal [ULN], alanine aminotransferase or aspartate aminotransferase≥2×ULN)

▪ Renal dysfunction (creatinine≥2 mg/dL creatinine clearance rate< 30 mL/min)

7. In the pregnancy or lactation period

8. With any conditions not suitable for the trial (investigators' decision)

# 4.2 Withdrawal criteria

Subjects are free to withdraw consent and discontinue participation in the study at any time and without prejudice to future treatment. A subject's participation in the study may be discontinued at any time at the investigator's discretion. Justifiable reasons for a subject to be withdrawn from the study include:

1. Inability to fully comply with the study protocol

2. Unacceptable toxicity

3. Best interest of the subject based upon the investigator’s discretion

4. At the request of the study subject at any time and for any reason

Subjects will be followed up unless the informed consent is withdrawn. The reason for withdrawal from study participation and the date must be documented in the case report form (CRF). The investigator must complete the last visit, including vital signs, physical examination, laboratory tests, disease status and AE assessment, all of which must be documented in the CRF.

# 5 Study procedures

# 5.1 Screening

Subjects aged 18-60 with AML who are willing to undergo haplo-HCT will be screened for eligibility. Medical history evaluation, vital signs, physical examination, ECOG performance status, PB and urine sampling for laboratory tests, electrocardiogram, chest imaging examination as well as BM assessment will be performed to determine study eligibility, all of which must be performed no more than 7 days before randomization.

# 5.2 Treatment allocation and blinding

Upon completion of all the required screening assessments, eligible subjects will be randomized at a 1:1 ratio to receive haplo-cord HCT or haplo-HCT. Randomization is performed with permuted blocks (block size four) and implemented through an interactive web-based response system. This is an open-label study. Participants and investigators are aware of treatment allocation.

# 5.3 Study treatment

# 5.3.1 Haplo-cord HCT group

Patients received Bu/Cy-based regimen. The UCB unit is infused 8 hours before the haploidentical graft infusion.

# 5.3.2 Haplo-HCT group

Patients received Bu/Cy-based regimen. Patients received haploidentical graft infusion alone (no UCB unit is infused).

# 5.4 Follow-up

Disease assessment including PB and BM assessment will be regularly performed posttransplantation. Routine blood is monitored before randomization, twice a week for the first month after enrolment, once a week from the 2nd to 3rd month after enrolment, once every two weeks from the 4th to 6th month after enrolment, once every month from the 7th to 24th month, and then once every three months until the study is completed. BM assessment is performed before randomization, every month for the first six months posttransplantation, every three months from the 6th to 24th month posttransplantation, and then every six months until the study is completed. Assessment of PB and BM are repeated when clinically indicated. Participants’ agreements to the additional collection of PB samples for immune reconstitution detection are supplementary asked.

Safety and tolerability will be assessed in all subjects within 2 years posttransplantation. Except for GVHD, all AEs are graded according to CTCAE version 4.0. aGVHD and cGVHD are graded as AEs according to our self-defined criteria.

# 6. Efficacy Assessments

# 6.1 Definitions

▪ Relapse is defined as either at least 5% of blasts in BM, the reappearance of blasts in PB or the development of extramedullary disease.

▪ Complete remission (CR) is defined as BM blasts<5%; absence of circulating blasts and blasts with Auer rods; absence of extramedullary disease; ANC≥1.0×10^9^/L and PLT≥100×10^9^/L.

▪ Partial remission (PR) is defined as 5%-20% blasts with or without extramedullary leukemia.

▪ No evidence of response (NR) is defined as a failure to obtain PR or better.

# 6.2 Primary Efficacy Endpoint

The 3-year OS is defined as the time from randomization until death from any cause or last follow-up.

# 6.3 Secondary Efficacy Endpoint

▪ PFS is defined as survival in continuous CR without relapse and refers to the time from randomization until relapse or death from any cause or last follow-up.

▪ CIR is defined as the time from randomization until death considered relapse-related.

▪ NRM is defined as the time from randomization until death considered transplantation-related without relapse.

# 6.4 Schedule and Methods of Efficacy Assessments

Disease assessment including routine blood and BM assessment will be regularly performed posttransplantation. Routine blood is monitored before randomization, twice a week for the first month after enrolment, once a week from the 2nd to 3rd month after enrolment, once every two weeks from the 4th to 6th month after enrolment, once every month from the 7th to 24th month, and then once every three months until the study is completed. BM assessment is performed before randomization, every month for the first six months posttransplantation, every three months from the 6th to 24th month posttransplantation, and then every six months until the study is completed. Assessment of PB and BM are repeated when clinically indicated. All subjects who complete treatment or withdraw from the study must receive an efficacy assessment.

# 7. Safety Evaluation

All subjects enrolled in the safety population (patients who received graft infusion) will be evaluated for safety and tolerability. Safety and tolerability will be assessed with vital signs, physical examination, clinical symptoms, and clinical laboratory evaluations (hematology, serum chemistry, urinalysis, electrocardiogram, and chest imaging examination). Except for GVHD, all AEs will be evaluated within 2 years posttransplantation according to CTCAE version 4.0. aGVHD and cGVHD are graded as AEs according to our self-defined criteria.

# 7.1 Medical History

Each subject's medical history must be obtained at screening. Sex, date of birth and information on any prior or existing medical conditions will be recorded on the appropriate CRF.

# 7.2 Vital Signs and Physical Examination

Vital signs and results of physical examination must be documented before randomization, twice a week for the first month after enrolment, once a week from the 2nd to 3rd month after enrolment, once every two weeks from the 4th to 6th month after enrolment, once every month from the 7th to 24th month, and then once every three months until the study is completed.

The next 6 items must be performed:

▪ Heart rate

▪ Blood pressure

▪ Body temperature

▪ Rate of respiration

▪ Physical examination

▪ ECOG performance status

# 7.3 Clinical Symptoms

During the study, the clinical symptoms of the patients must be documented.

# 7.4 Clinical Laboratory Evaluations

Before the initiation of the study, the monitors will document the normal range of each test in every involved laboratory. During the study, the following items must be performed:

▪ Routine blood: white cell counts, neutrophil cell counts, hemoglobin, and platelet counts

▪ Hepatic function: total bilirubin (both direct bilirubin and indirect bilirubin must be documented when the total bilirubin elevates), ALT, AST, lactic dehydrogenase, alkaline phosphatase, albumin and total protein

▪ Renal function: serum creatinine, urea nitrogen and uric acid

▪ Electrolytes: sodium, potassium, calcium and magnesium

▪ Coagulation function: prothrombin time, prothrombin time-international normalized ratio, activated partial thromboplastin time and fibrinogen

▪ Urinalysis: protein, glucose and erythrocyte

▪ Electrocardiogram

▪ Chest imaging examination

# 8. Adverse Events and Serious Adverse Events

The investigator is responsible for detecting, documenting and reporting events that meet the definition of an adverse event (AE) or serious adverse event (SAE).

# 8.1 Definitions

# 8.1.1 Adverse Events

An AE is any untoward medical occurrence in a subject of a clinical investigation, which does not necessarily have a causal relationship to the medicinal product. Therefore, an AE can be any unfavorable and unintended sign, including an abnormal laboratory finding, symptom, or disease (new or exacerbated), whether or not it is considered to be related to the product. This definition includes any newly occurring event or previous condition that has increased in severity or frequency since the administration of the product. However, relapse or death due to relapse should not be recorded as AEs.

# 8.1.2 Serious Adverse Events

A serious adverse event is any untoward medical occurrence that, at any dose:

▪ Results in death

▪ Is life-threatening

▪ Requires hospitalization or prolongation of existing hospitalization, i.e., the AE requires at least a 24-hour inpatient hospitalization or prolongs a hospitalization beyond the expected length of stay.

▪ Hospitalization or prolongation of existing hospitalization for social reasons will not be reported as an SAE.

▪ Results in disability/incapacity

▪ Congenital anomaly/birth defect

▪ Important medical event

Medical or scientific judgment should be exercised in deciding whether SAE reporting is appropriate in other situations. An important medical event is an event that may not result in death, be life-threatening, or require hospitalization, but is clearly of major clinical significance. The AE may jeopardize the subject or require intervention to prevent a serious outcome.

# 8.2 Assessment of Severity

With the exception of GVHD, all AEs are graded according to CTCAE version 4.0. When CTCAE version 4.0 criteria do not apply, the severity will be defined according to the following criteria:

| **Severity** | **Description** |
| --- | --- |
| Grade 1- Mild | Asymptomatic or mild symptoms; clinical or diagnostic observations only; intervention not indicated |
| Grade 2- Moderate | Minimal, local or noninvasive intervention indicated; limiting age-appropriate instrumental activities of daily living (ADL) |
| Grade 3- Severe | Medically significant but not immediately life-threatening; hospitalization or prolongation of hospitalization indicated; disabling; limiting self-case ADL |
| Grade 4- Life-threatening | Life-threatening consequences; urgent intervention indicated |
| Grade 5- Death | Death |

In this study, aGVHD is graded according to the 1994 Consensus Conference on Acute GVHD Grading, and cGVHD is graded according to the National Institutes of Health (NIH) criteria.19,20 aGVHD and cGVHD are graded as AEs according to our self-defined criteria. Grade I aGVHD without intervention is graded as grade 1 AE, grade I aGVHD with intervention as grade 2 AE, grade II aGVHD as grade 3 AE, grade III-IV aGVHD as grade 4 AE, and death due to aGVHD as grade 5 AE. With regards to cGVHD, mild cGVHD without systematic intervention is graded as grade 1 AE, mild cGVHD with systematic intervention as grade 2 AE, moderate cGVHD as grade 3 AE, severe cGVHD as grade 4 AE, and death due to cGVHD as grade 5 AE.

# 8.3 Assessment of Causality

The investigator must determine the relationship of each AE and SAE to study treatment. The relationship of an AE or SAE to study treatment will be defined according to the following criteria:

▪ Definite: There is a clear temporal relationship to study treatment, with no other possible cause.

▪ Possible: A temporal relationship to study treatment is not clear, and alternative etiologies are possible.

▪ Not related: There is no temporal relationship to study treatment, and/or there is evidence of an alternative cause such as a concurrent medication or illness.

# 8.4 Recording and Reporting Adverse Events and Serious Adverse Events

All AEs and SAEs except hematologic AEs must be recorded in the appropriate CRF, whether or not they are associated to be causally related to the study treatment. Each SAE must be reported promptly on the Serious Adverse Event Report Form, and submitted to the Independent Ethics Committee within 24 hours by the investigator. The information recorded on the Serious Adverse Event Report Form will include at least the following: subject number, identity of the event, study drug name and dose, investigator's assessment of the event's severity and relationship to study treatment, and investigator's name and signature. Clinical monitors must collect and verify detailed information about AEs and SAEs when examining original medical records. All AEs and SAEs should be followed up until resolved.

# 9. Rules of Withdrawal

# 9.1 Subjects Withdraw from the Study

Subjects can withdraw from the study at any time for any reason without impact on the investigator’s right to treat disease for subjects. Based upon the interest of subjects, the investigator has the right to request subjects to withdraw from the study for any reason including concomitant disease, AEs or treatment failure. The core group of clinical study reserves the right to request subjects to withdraw from the study for deviation(s) from the protocol, administrative reasons, or other effective or ethical reasons.

The last assessment for subjects must be performed and documented in the CRF regardless of the time and reason for withdrawal. The reason for withdrawal from study participation must be documented in the CRF. All documents related to subjects should be completed. Despite the withdrawal from the study, those subjects should be followed up and documented about their diseases until the withdrawal of informed consent.

For subjects who withdraw from the study due to concomitant diseases or AEs, the details must be documented in the CRF with other appropriate and valuable data attached.

# 9.2 Premature Termination of the Study

Reasons for premature termination of the study include external events, repetition of SAEs, growing incidence of treatment-related death and slow enrolment in the study. All subjects will be informed of the premature termination of the study by written consent. Any subjects who decide to discontinue participating in the study must report to the principal investigator.

# 10. Rules of Follow-up

# 10.1 Follow-up Period

Starting from randomization.

# 10.2 Visit Scheduling

First day at transplantation, twice a week for the posttransplantation, once a week from the 2nd to 3rd month, once every two weeks from the 4th to 6th month, once every month from the 7th to 24th month, and then once every three months until the study is completed (detailed in 16.1 Monitoring Schema).

# 10.3 Contents

The contents of every follow-up visit include complaints of subjects, vital signs, physical examination, clinical symptoms and clinical laboratory evaluations (hematology, serum chemistry, urinalysis, electrocardiogram, chest imaging examination, and BM assessment). All of the results must be documented in the original medical record.

# 11. Data Analysis and Statistical Considerations

# 11.1 Hypotheses

The primary endpoint is the 3-year OS. The null and alternative hypotheses are designed to demonstrate the superiority of haplo-cord HCT over haplo-HCT with respect to OS. Superiority will be determined using the following hypothesis:

H0: OS with haplo-cord HCT≤OS with haplo-HCT

H1: OS with haplo-cord HCT>OS with haplo-HCT

# 11.2 Study Design Considerations

This prospective, open-label, multicenter, randomized, phase 3 study compares the efficacy of haplo-cord HCT and haplo-HCT in AML. The primary outcome is the 3-year OS, and the study is designed to determine if haplo-cord HCT is superior to haplo-HCT in the study population with respect to OS. Based on the retrospective findings in 51 haplo-cord HCT and 87 haplo-HCT AML patients treated in the First Affiliated Hospital of Soochow University between 2011-2013, the 3-year OS for haplo-HCT patients is 60.1%, and a clinically meaningful improvement in 3-year OS would be 17.2% for those who receive haplo-cord HCT.

# 11.2.1 Sample Size Assumptions

The sample size calculation is based on the primary endpoint, the 3-year OS, with the following assumptions:

▪ The 3-year OS in the haplo-HCT group: 60.1%

▪ The 3-year OS in the haplo-cord HCT group: 77.3%

▪ A 1:1 randomization scheme

▪ A 5% one-tailed risk of erroneously claiming a difference in the presence of no true underlying difference (alpha)

▪ A 90% chance of successfully declaring a difference in the presence of a true underlying difference (power)

▪ Uniform or equal in accrual pattern

▪ A 6% percent of cases drop in each group

Under the above assumptions, a sample size of 134 subjects in each group is required.

# 11.2.2 Primary Efficacy Endpoint

The primary efficacy endpoint is the 3-year OS.

# 11.2.3 Secondary Efficacy Endpoints

The secondary efficacy endpoints include PFS, CIR, and NRM.

# 11.3 Data Analysis Considerations

# 11.3.1 Analysis Population

The primary population will be the intent-to-treat (ITT) population, which is defined as all subjects randomized to the two groups. ITT population will be the basis for the analysis of efficacy endpoints in this study. Randomly assigned patients who received graft infusion will be included in the modified intention-to-treat (mITT) population. The safety population will be the patients who received graft infusion.

# 11.3.2 Analysis Plan

# 11.3.2.1 Baseline Data

Baseline characteristics will be summarized and described in a frequency list.

# 11.3.2.2 Analysis of Efficacy

The definition of efficacy endpoints has been detailed in the previous section. CIR and NRM are calculated by accounting for competing risks. NRM is a competing risk for CIR, and relapse is a competing risk for NRM. The comparison of the cumulative incidence is done using Gray's test and Fine and Gray's model. OS and DFS are estimated using the Kaplan‒Meier method and compared using the log-rank test. The corresponding hazard ratio (HR) and 95% CI are estimated using the Cox proportional hazards model. All statistical tests are two-tailed with a significance level of 0.05. SPSS (SPSS Inc., Chicago, IL, USA) and R (R Development Core Team, Vienna, Austria) are used for all data analysis.

# 11.3.2.3 Analysis of Safety

Safety and tolerability will be assessed by the incidence and severity of AEs, including laboratory test values, physical examination, vital signs, and ECOG performance scores. The definition of AEs has been detailed in the previous section. Except for GVHD, all AEs are graded according to CTCAE version 4.0. aGVHD and cGVHD are graded as AEs according to our self-defined criteria. All subjects will be monitored for AEs within 2 years posttransplantation. Categorical data will be summarized by the proportion of total subjects. Quantitative data will be described using arithmetic average or median for central tendency and standard deviation or interquartile range for distribution range.

# 12. Materials for the Study

All materials provided to study sites and investigators are as follows:

▪ The study protocol

▪ Informed consent

▪ CRF

# 13. Ethical Considerations

# 13.1 Responsibility of Investigators

The investigators have the responsibility for guaranteeing the clinical study’s compliance with the protocol, Chinese good clinical practice (GCP) guidelines and applicable laws and regulations.

# 13.2 Informed Consent Process

Before participation in the study, subjects must be informed about the objectives, methods, possible benefits, potential risks and possible discomforts of the study by investigators. They also should be informed that participation in the study would be voluntary, they can withdraw from the study at any time, there is no impact on the treatment of the disease whether they take part in the study and their privacy will be protected.

Subjects or their legally acceptable representative should have enough time to read the informed consent and raise queries. Written informed consent must be obtained from each subject, or their legally acceptable representative.

# 13.3 Good Clinical Practice

This study will be conducted following the Declaration of Helsinki and Chinese GCP. The study will be conducted only if it is approved by the ethical review committee of the principal study site. The investigators will guarantee that the study will be conducted following applicable laws and regulations, and scientific and ethical principles of the People’s Republic of China. If the protocol needs revision during the study, the revised version must be reapproved by the ethical review committee of the principal study site. If new data related to study treatment are discovered, the informed consent must be revised and the revision must be reapproved by the ethical review committee of the principal study site and subjects.

# 13.4 Protection of Subjects’ Personal Data

Data collected in the study are limited to the efficacy and safety related to the study treatment. Data will be collected and used in accordance with applicable laws and regulations.

# 14. Administrative Requirements

Neither the investigator nor the applicant can revise the protocol without the agreement of the opposite side. All revisions of the protocol must be released by the applicant institution. To ensure the integrity, accuracy and reliability of the data, relevant results of examination and treatment must be documented in the original medical record and CRF. Independent clinical monitoring is performed regularly by a panel of qualified and experienced study investigators composed of hematologists who are blinded as to the treatment assignments.

# 15. References

1. Wang, Y. *et al.* Haploidentical vs identical-sibling transplant for AML in remission: a multicenter, prospective study. *Blood* **125**, 3956-62 (2015).

2. De Kouchkovsky, I. & Abdul-Hay, M. 'Acute myeloid leukemia: a comprehensive review and 2016 update'. *Blood Cancer J* **6**, e441 (2016).

3. Döhner, H., Weisdorf, D.J. & Bloomfield, C.D. Acute Myeloid Leukemia. *N Engl J Med* **373**, 1136-52 (2015).

4. Stölzel, F. *et al.* Karyotype complexity and prognosis in acute myeloid leukemia. *Blood Cancer J* **6**, e386 (2016).

5. Ravandi, F. *et al.* Phase I/II study of combination therapy with sorafenib, idarubicin, and cytarabine in younger patients with acute myeloid leukemia. *J Clin Oncol* **28**, 1856-62 (2010).

6. Ravandi, F. *et al.* Final report of phase II study of sorafenib, cytarabine and idarubicin for initial therapy in younger patients with acute myeloid leukemia. *Leukemia* **28**, 1543-5 (2014).

7. Ravandi, F. *et al.* Phase 2 study of azacytidine plus sorafenib in patients with acute myeloid leukemia and FLT-3 internal tandem duplication mutation. *Blood* **121**, 4655-62 (2013).

8. Yafour, N. *et al.* How to prevent relapse after allogeneic hematopoietic stem cell transplantation in patients with acute leukemia and myelodysplastic syndrome. *Curr Res Transl Med* **65**, 65-69 (2017).

9. Wang, Y. *et al.* Haploidentical transplant for myelodysplastic syndrome: registry-based comparison with identical sibling transplant. *Leukemia* **30**, 2055-2063 (2016).

10. Gluckman, E. *et al.* Milestones in umbilical cord blood transplantation. *Br J Haematol* **154**, 441-7 (2011).

11. Brunstein, C.G. *et al.* Allogeneic hematopoietic cell transplantation for hematologic malignancy: relative risks and benefits of double umbilical cord blood. *Blood* **116**, 4693-9 (2010).

12. Chen, J. *et al.* Combination of a haploidentical SCT with an unrelated cord blood unit: a single-arm prospective study. *Bone Marrow Transplant* **49**, 206-11 (2014).

13. Taskinen, M.H., Huttunen, P., Niittyvuopio, R. & Saarinen-Pihkala, U.M. Coinfusion of mobilized hematopoietic stem cells from an HLA-mismatched third-party donor with umbilical cord blood graft to support engraftment. *J Pediatr Hematol Oncol* **36**, e518-23 (2014).

14. van Besien, K. & Childs, R. Haploidentical cord transplantation-The best of both worlds. *Semin Hematol* **53**, 257-266 (2016).

15. Bautista, G. *et al.* Cord blood transplants supported by co-infusion of mobilized hematopoietic stem cells from a third-party donor. *Bone Marrow Transplant* **43**, 365-73 (2009).

16. Sebrango, A. *et al.* Haematopoietic transplants combining a single unrelated cord blood unit and mobilized haematopoietic stem cells from an adult HLA-mismatched third party donor. Comparable results to transplants from HLA-identical related donors in adults with acute leukaemia and myelodysplastic syndromes. *Best Pract Res Clin Haematol* **23**, 259-74 (2010).

17. Liu, H. *et al.* Reduced-intensity conditioning with combined haploidentical and cord blood transplantation results in rapid engraftment, low GVHD, and durable remissions. *Blood* **118**, 6438-45 (2011).

18. Tian, H., Qu, Q., Liu, L. & Wu, D. Advances in Stem Cell Therapy for Leukemia. *Curr Stem Cell Res Ther* **11**, 158-65 (2016).

19. Przepiorka, D. *et al.* 1994 Consensus Conference on Acute GVHD Grading. *Bone Marrow Transplant* **15**, 825-8 (1995).

20. Jagasia, M.H. *et al.* National Institutes of Health Consensus Development Project on Criteria for Clinical Trials in Chronic Graft-versus-Host Disease: I. The 2014 Diagnosis and Staging Working Group report. *Biol Blood Marrow Transplant* **21**, 389-401.e1 (2015).

# 16. Appendices

# 16.1 Monitoring Schema

|  | **Screen** | **d01** | **+3d** | **+1w** | **+10d** | **+2w** | **+17d** | **+3w** | **+24d** |
| --- | --- | --- | --- | --- | --- | --- | --- | --- | --- |
| **PE** | + | + | + | + | + | + | + | + | + |
| **ECOG** | + | + | + | + | + | + | + | + | + |
| **PB** | + | + | + | + | + | + | + | + | + |
| **HF** | + | + | + | + | + | + | + | + | + |
| **RF** | + | + | + | + | + | + | + | + | + |
| **CMV** | + | - | + | + | + | + | + | + | + |
| **EBV** | + | - | + | + | + | + | + | + | + |
| **EM** | + | - | + | + | + | + | + | + | + |
| **BM** | + | - | - | - | - | - | - | - | - |
|  | **+1m** | **+5w** | **+6w** | **+7w** | **+2m** | **+9w** | **+10w** | **+11w** | **+3m** |
| **PE** | + | + | + | + | + | + | + | + | + |
| **ECOG** | + | + | + | + | + | + | + | + | + |
| **PB** | + | + | + | + | + | + | + | + | + |
| **HF** | + | + | + | + | + | + | + | + | + |
| **RF** | + | + | + | + | + | + | + | + | + |
| **CMV** | + | + | + | + | + | + | + | + | + |
| **EBV** | + | + | + | + | + | + | + | + | + |
| **EM** | + | + | + | + | + | + | + | + | + |
| **BM** | + | - | - | - | + | - | - | - | + |
|  | **+14w** | **+4m** | **+18w** | **+5m** | **+22w** | **+6m** | **+7m** | **+8m** | **+9m** |
| **PE** | + | + | + | + | + | + | + | + | + |
| **ECOG** | + | + | + | + | + | + | + | + | + |
| **PB** | + | + | + | + | + | + | + | + | + |
| **HF** | + | + | + | + | + | + | + | + | + |
| **RF** | + | + | + | + | + | + | + | + | + |
| **CMV** | + | + | + | + | + | + | + | + | + |
| **EBV** | + | + | + | + | + | + | + | + | + |
| **EM** | + | + | + | + | + | + | + | + | + |
| **BM** | - | + | - | + | - | + | - | - | + |
|  | **+10m** | **+11m** | **+1y** | **+13m** | **+14m** | **+15m** | **+16m** | **+17m** | **+18m** |
| **PE** | + | + | + | + | + | + | + | + | + |
| **ECOG** | + | + | + | + | + | + | + | + | + |
| **PB** | + | + | + | + | + | + | + | + | + |
| **HF** | + | + | + | + | + | + | + | + | + |
| **RF** | + | + | + | + | + | + | + | + | + |
| **CMV** | + | + | + | + | + | + | + | + | + |
| **EBV** | + | + | + | + | + | + | + | + | + |
| **EM** | + | + | + | + | + | + | + | + | + |
| **BM** | - | - | + | - | - | + | - | - | + |
|  | **+19m** | **+20m** | **+21m** | **+22m** | **+23m** | **+2y** | **+27m** | **+30m** | **+33m** |
| **PE** | + | + | + | + | + | + | + | + | + |
| **ECOG** | + | + | + | + | + | + | + | + | + |
| **PB** | + | + | + | + | + | + | + | + | + |
| **HF** | + | + | + | + | + | + | + | + | + |
| **RF** | + | + | + | + | + | + | + | + | + |
| **CMV** | + | + | + | + | + | + | + | + | + |
| **EBV** | + | + | + | + | + | + | + | + | + |
| **EM** | + | + | + | + | + | + | + | + | + |
| **BM** | - | - | + | - | - | + | - | + | - |
|  | **+3y** | **+39m** | **+42m** | **+45m** | **+4y** | **+51m** | **+54m** | **+57m** | **+5y** |
| **PE** | + | + | + | + | + | + | + | + | + |
| **ECOG** | + | + | + | + | + | + | + | + | + |
| **PB** | + | + | + | + | + | + | + | + | + |
| **HF** | + | + | + | + | + | + | + | + | + |
| **RF** | + | + | + | + | + | + | + | + | + |
| **CMV** | + | + | + | + | + | + | + | + | + |
| **EBV** | + | + | + | + | + | + | + | + | + |
| **EM** | + | + | + | + | + | + | + | + | + |
| **BM** | + | - | + | - | + | - | + | - | + |

PE=physical examination, including heart rate, blood pressure, body temperature, rate of respiration and other physical examination; ECOG=Eastern Cooperative Oncology Group performance status; PB=count of blood cells in peripheral blood; HF=hepatic function; RF=renal function; EM=extramedullary leukemia evaluation; BM=bone marrow test.

# 16.2 Appendix 1 Diagnosis and Classification of aGVHD and cGVHD

**Grading of aGVHD**

| **Grade** |  | **Degree of organ involvement** |
| --- | --- | --- |
| I |  | Stage 1-2 skin rash; no gut involvement; no liver involvement; no decrease in clinical performance |
| II |  | Stage 1-3 skin rash; stage 1 gut involvement or stage 1 liver involvement (or both); mild decrease in clinical performance |
| III |  | Stage 2-3 skin rash; stage 2-3 gut involvement or 2-4 liver involvement (or both); marked decrease in clinical performance |
| IV |  | Similar to Grade III with stage 2-4 organ involvement and extreme decrease in clinical performance |

Przepiorka D, *et al*. 1994 Consensus Conference on Acute GVHD Grading. *Bone Marrow Transplant* **15**, 825-828 (1995).

**Grading of cGVHD**

| **NIH Global Severity of chronic GVHD** |
| --- |
| Mild chronic GVHD |
| 1 or 2 Organs involved with no more than score 1 plus Lung score 0 |
| Moderate chronic GVHD |
| 3 or More organs involved with no more than score 1 |
| OR |
| At least 1 organ (not lung) with a score of 2 |
| OR |
| Lung score 1 |
| Severe chronic GVHD |
| At least 1 organ with a score of 3 |
| OR |
| Lung score of 2 or 3 |
| Key points:  In skin: higher of the 2 scores to be used for calculating global severity.  In lung: FEV1 is used instead of clinical score for calculating global severity.  If the entire abnormality in an organ is noted to be unequivocally explained by a non-GVHD documented cause, that organ is not included for calculation of the global severity.  If the abnormality in an organ is attributed to multifactorial causes (GVHD plus other causes), the scored organ will be used for calculation of the global severity regardless of the contributing causes (no downgrading of organ severity score). |

Jagasia MH, *et al*. National Institutes of Health Consensus Development Project on Criteria for Clinical Trials in Chronic Graft-versus-Host Disease: I. The 2014 Diagnosis and Staging Working Group report. *Biol Blood Marrow Transplant* **21**, 389-401.e1 (2015).


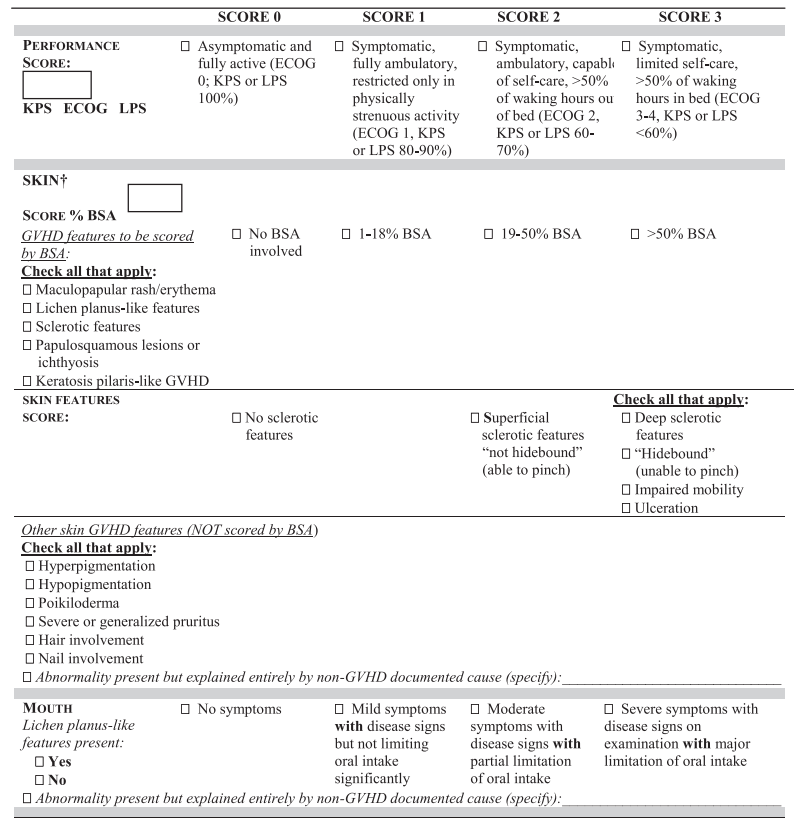


**Figure 1.**

Organ scoring of chronic GVHD. ECOG indicates Eastern Cooperative Oncology Group; KPS, Karnofsky Performance Status; LPS, Lansky Performance Status; BSA, body surface area; ADL, activities of daily living; LFTs, liver function tests; AP, alkaline phosphatase; ALT, alanine aminotransferase; ULN, normal upper limit. *Weight loss within 3 months. †Skin scoring should use both percentage of BSA involved by disease signs and the cutaneous features scales. When a discrepancy exists between the percentage of total body surface (BSA) score and the skin feature score, OR if superficial sclerotic features are present (Score 2), but there is impaired mobility or ulceration (Score 3), the higher level should be used for the final skin scoring. **Lung scoring should be performed using both the symptoms and FEV1 scores whenever possible. FEV1 should be used in the final lung scoring where there is discrepancy between symptoms and FEV1 scores.


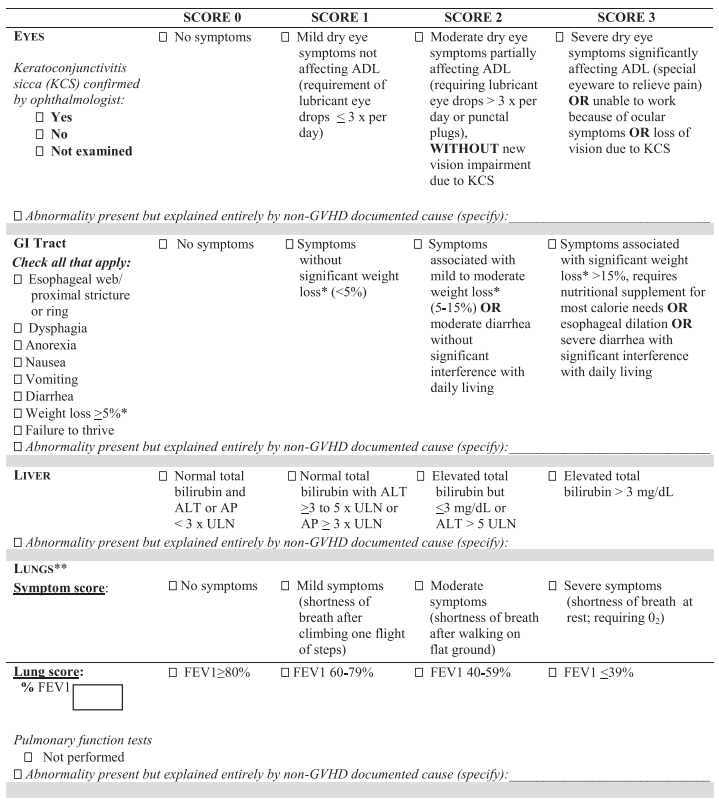


**Figure 1.** (continued).


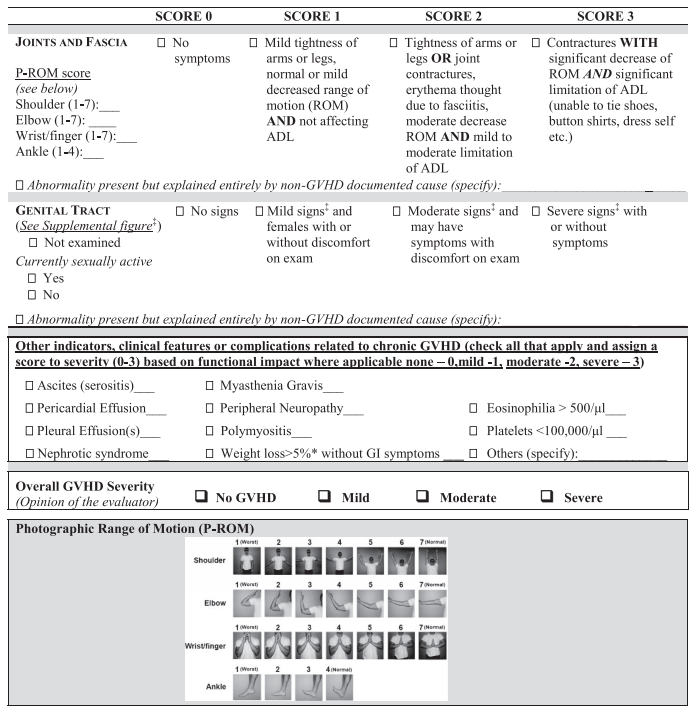


**Figure 1.** (continued).

Jagasia MH, *et al*. National Institutes of Health Consensus Development Project on Criteria for Clinical Trials in Chronic Graft-versus-Host Disease: I. The 2014 Diagnosis and Staging Working Group report. *Biol Blood Marrow Transplant* **21**, 389-401.e1 (2015).

# 16.3 Appendix 2 ECOG Performance Status

**ECOG Performance Status**

| **Grade** | **ECOG** |
| --- | --- |
| 0 | Fully active, able to carry on all pre-disease performance without restriction |
| 1 | Restricted in physically strenuous activity but ambulatory and able to carry out work of a light or sedentary nature, e.g., light house work, office work |
| 2 | Ambulatory and capable of all selfcare but unable to carry out any work activities. Up and about more than 50% of waking hours |
| 3 | Capable of only limited selfcare, confined to bed or chair more than 50% of waking hours |
| 4 | Completely disabled. Cannot carry on any selfcare. Totally confined to bed or chair |
| 5 | Dead |

Oken MM, *et al*. Toxicity and response criteria of the Eastern Cooperative Oncology Group. *Am* *J Clin Oncol* **5**, 649-655 (1982).
